# Harnessing the untapped potential of nucleotide‐binding oligomerization domain ligands for cancer immunotherapy

**DOI:** 10.1002/med.21557

**Published:** 2018-12-13

**Authors:** Sanja Nabergoj, Irena Mlinarič‐Raščan, Žiga Jakopin

**Affiliations:** ^1^ University of Ljubljana, Faculty of Pharmacy Ljubljana Slovenia

**Keywords:** adjuvants, cancer immunotherapy, immunotherapeutics, NOD1 agonists, NOD1 antagonists, NOD2 agonists, NOD2 antagonists

## Abstract

In the last decade, cancer immunotherapy has emerged as an effective alternative to traditional therapies such as chemotherapy and radiation. In contrast to the latter, cancer immunotherapy has the potential to distinguish between cancer and healthy cells, and thus to avoid severe and intolerable side‐effects, since the cancer cells are effectively eliminated by stimulated immune cells. The cytosolic nucleotide‐binding oligomerization domains 1 and 2 receptors (NOD1 and NOD2) are important components of the innate immune system and constitute interesting targets in terms of strengthening the immune response against cancer cells. Many NOD ligands have been synthesized, in particular NOD2 agonists that exhibit favorable immunostimulatory and anticancer activity. Among them, mifamurtide has already been approved in Europe by the European Medicine Agency for treating patients with osteosarcoma in combination with chemotherapy after complete surgical removal of the primary tumor. This review is focused on NOD receptors as promising targets in cancer immunotherapy as well as summarizing current knowledge of the various NOD ligands exhibiting antitumor and even antimetastatic activity in vitro and in vivo.

**Abbreviations**
ActD: actinomycin D
AP‐1: activator protein 1
AOM: azoxymethane
ASC: apoptosis‐associated speck‐like protein containing caspase recruitment domain
ATG16L1: autophagy‐related 16‐like 1
Bid: BH3‐interacting domain death agonist
CARD: caspase recruitment domain
CCL2: chemokine ligand 2
CDDP: cisplatin
cIAP: cellular inhibitor of apoptosis protein
CRC: colorectal cancer
CSF: colony‐stimulating factor
CTX: cyclophosphamide
DAMP: danger‐associated molecular pattern
DC: dendritic cell
DMPG: dimyristoylphosphatidylglycerol
DOX: doxorubicin
DSPC: distearoylphosphatidylcholine
DSS: dextran sulfate sodium
DTX: docetaxel
ERK: extracellular signal‐regulated kinase
FCA: Freund's complete adjuvant
GC: gastric cancer
GDP: glycerol dipalmitate
HNSCC: head and neck squamous cell carcinoma
IAP: inhibitor of the apoptosis
ICAM‐1: intercellular adhesion molecule‐1
ICD: immunogenic cell death
iE‐DAP: d‐glutamyl‐*meso*‐diaminopimelic acid
IFN: interferon
IFO: ifosfamide
IKK: IκB kinase
IκB: protein inhibitor of NF‐κB
IL: interleukin
IRF: interferon regulatory factor
JNK: c‐Jun N‐terminal kinase
LLC: Lewis lung carcinoma
LPS: lipopolysaccharide
LRR: leucine‐rich repeat
mAb: monoclonal antibody
MAPK: mitogen‐associated protein kinase
MAVS: mitochondrial antiviral signaling
MBSA: maleylated BSA
MDP: muramyl dipeptide
MDSC: myeloid‐derived suppressor cell
MMP: matrix metalloproteinase
MoDC: monocyte‐derived dendritic cell
MTX: methotrexate
NACHT: nucleotide‐binding domain
NEMO: nuclear factor κB essential modulator
NLR: nucleotide‐binding oligomerization domain‐like receptor
NK: natural killer
NLRP: NACHT‐, LRR‐, and PYD‐domain–containing protein
NOD: nucleotide‐binding oligomerization domain
OSCC: oral squamous cell carcinoma
PAMP: pathogen‐associated molecular pattern
PBMC: peripheral blood mononuclear cell
PC: pancreatic cancer
PGN: peptidoglycan
PROK2: prokineticin 2
PRR: pattern‐recognition receptor
PS: phosphatidylserine
PTX: paclitaxel
RIPK2: receptor‐interacting serine/threonine‐protein kinase 2
PolyG: polyguanylic acid
SAR: structure‐activity relationship
STAT1: signal transducer and activator of transcription 1
S100A8: S100 calcium‐binding protein A8
TAB: transforming growth factor binding protein
TAK1: transforming growth factor β‐activated kinase 1
TBK1: TRAF‐associated nuclear factor‐κB activator–binding kinase 1
TGF: transforming growth factor
Th1: type 1T helper
TIMP1: tissue inhibitor of metalloproteinase 1
TLR: Toll‐like receptor
TME: tumor microenvironment
TNF‐α: tumor necrosis factor α
TRAF: tumor necrosis factor receptor–associated factor
Tri‐DAP: l‐alanyl‐γ‐d‐glutamyl‐*meso*‐DAP
XIAP: X‐linked inhibitor of apoptosis protein

## 1 INTRODUCTION

**Figure 1:**
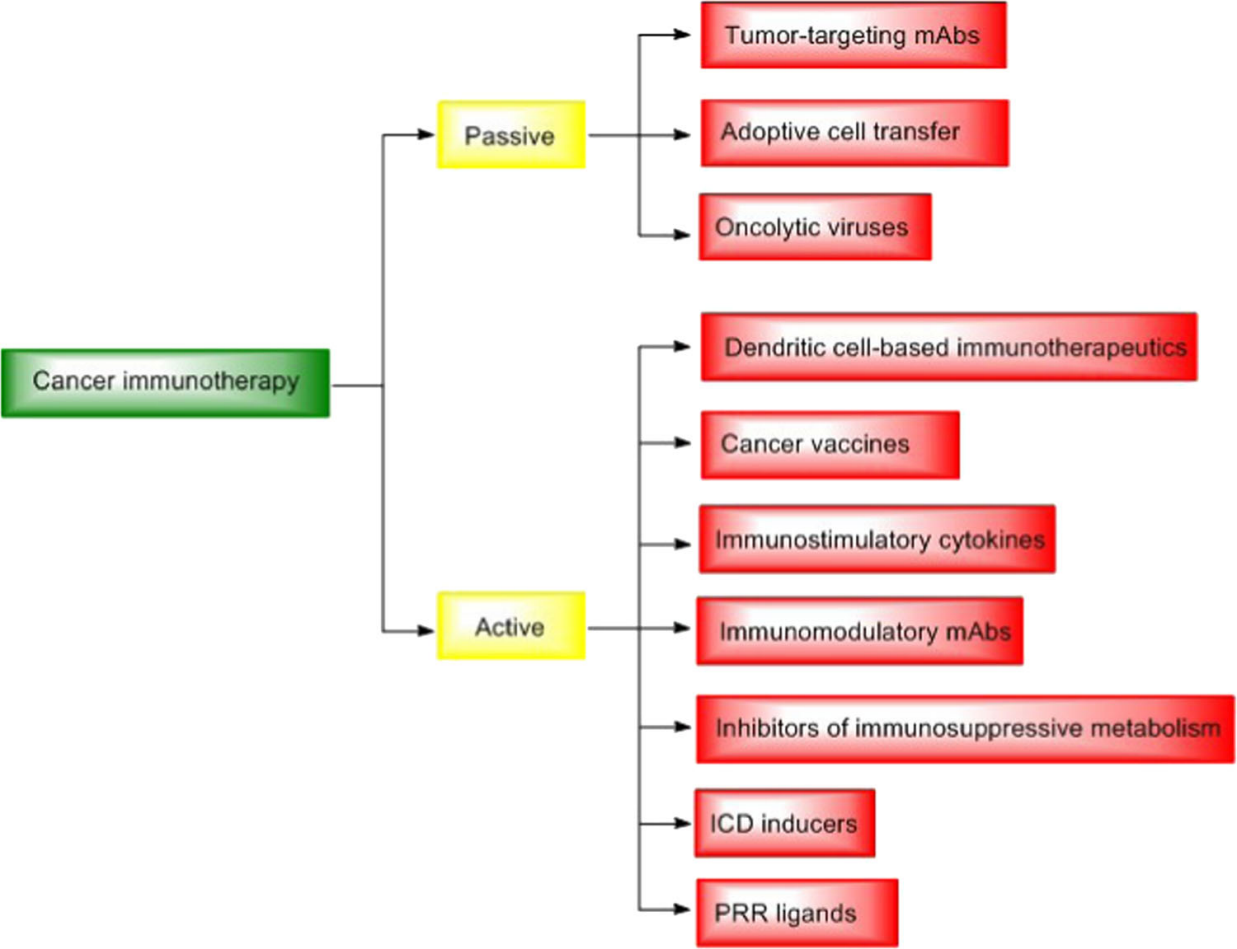
Cancer immunotherapies. Cancer immunotherapies are broadly classified as passive or active, based on their ability to activate host's immune system against cancer cells. The group of passive immunotherapeutics consist of tumor‐targeting mAbs, adoptive cell transfer, and oncolytic viruses, while active immunotherapeutics comprise dendritic cell‐based immunotherapeutics, cancer vaccines, immunostimulatory cytokines, immunomodulatory mAbs, inhibitors of immunosuppressive metabolism, ICD inducers, and PRR ligands. ICD, immunogenic cell death; mAbs, monoclonal antibodies; PRR, pattern‐recognition receptor [Color figure can be viewed at wileyonlinelibrary.com]

The connection between the immune system and cancer was first proposed by Rudolph Virchow in 1863 when he observed the presence of leukocytes in neoplastic tissues.^1^ A few years later, in 1891, William Coley laid the groundwork for modern immunotherapy with the discovery that the use of a bacterial vaccine in treating primarily inoperable sarcoma resulted in a cure rate greater than 10%.^2^ A similar attempt was made in 1976 when Morales et al^3^ used BCG, a live attenuated strain of bacteria *Mycobacterium bovis*, for treating bladder cancer. In fact, BCG injection still represents the main intravesical immunotherapy for treating superficial bladder cancer.^4^ The field of cancer immunotherapy has been intensively developed over the last decade as evidenced by an increasing number of newly approved immunotherapeutics for human use.^5^ In general, cancer immunotherapeutics are classified as passive or active, based on their ability to activate the host's immune system against cancer cells. Passive immunotherapeutics are designed to act like certain components of the host's immune system whereas active immunotherapeutics stimulate the host's immune system to induce its own response against cancer cells.^6^, ^7^ The group of passive immunotherapeutics comprises tumor‐targeting monoclonal antibodies (mAbs), adoptive cell transfer and oncolytic viruses, while active immunotherapeutics comprise dendritic cell‐based immunotherapeutics, cancer vaccines, immunostimulatory cytokines, immunomodulatory mAbs, inhibitors of immunosuppressive metabolism, immunogenic cell death (ICD) inducers, and pattern‐recognition receptor (PRR) ligands (Figure 1).^6^, ^7^ The latter group, in particular, has a lot of untapped potential for use as an adjunct to current cancer therapies, as evidenced by recent discoveries.^8^

PRRs are evolutionarily conserved proteins in the innate immune system that are involved in the recognition of various pathogen‐ or danger‐associated molecular patterns (PAMPs and DAMPs).^9^ They are subdivided into five families including Toll‐like receptors (TLRs), RIG‐I‐like receptors, NOD‐like receptors (NLRs), AIM2‐like receptors and C‐type lectins.^10^ NOD1 and NOD2 are the most studied members of the NLR family and sense conserved peptidoglycan (PGN) fragments found in bacterial cell walls. NOD1 senses *meso*‐diaminopimelic acid (DAP)‐containing fragments of PGN, such as the d‐glutamyl‐*meso*‐diaminopimelic acid (iE‐DAP) and l‐alanyl‐γ‐d‐glutamyl‐*meso*‐DAP (Tri‐DAP) found in Gram‐negative bacteria and certain Gram‐positive bacteria. NOD2, however, is activated by *N*‐(acetylmuramyl)‐l‐alanyl‐d‐isoglutamine (muramyl dipeptide [MDP]), a small PGN fragment found in various Gram‐negative bacteria and Gram‐positive bacteria.^11^, ^12^, ^13^, ^14^ Due to their ability to detect bacterial PGN and, consequently, to activate an inflammatory response, NODs are considered as key molecules in host defense and inflammation.^15^ In humans, for example, mutations in genes encoding NOD2 are associated with inflammatory diseases including Blau syndrome and early onset sarcoidosis^16^, ^17^ as well as with increased risk of developing Crohn's disease.^18^ Despite the well‐known role of NODs in pathogen recognition and inflammation, they are also of great importance in the process of cancer development, as evidenced by findings that mutations in the genes encoding NODs are also associated with increased risk of several cancers.^15^, ^19^, ^20^ Moreover, NODs are also targets of interest in terms of strengthening the immune response against cancer cells. Specifically, NOD agonists possess the ability to stimulate anticancer activity of immune cells, in particular monocytes and macrophages. A NOD2 agonist, mifamurtide, has already been approved in immunotherapy for patients with osteosarcoma, in combination with chemotherapy following complete surgical resection of the primary tumor. Other NOD agonists, as well as NOD antagonists, have been under investigation in preclinical and clinical studies.

The purpose of this review is, therefore, to evaluate NOD receptors as new targets in cancer immunotherapy and to highlight the NOD1 and NOD2 agonists as well as antagonists reported to exhibit anticancer activity.

## 2 NOD1 AND NOD2 PROTEINS—EXPRESSION, STRUCTURE, AND SIGNALING

### 2.1 Expression

NOD1 and NOD2 are intracellular proteins encoded by the *CARD4* gene found on chromosome 7p14‐15, and the *CARD15* gene found on chromosome 16q12.^21^ Both receptors are located in the cell cytosol and, in certain cells, also at the plasma membrane.^22^ Their recruitment to the cell membrane has been observed in various epithelial cells and recognized as a crucial event for activation of the NF‐κB signaling pathway following bacterial PGN binding.^23^, ^24^ Interestingly, although similar in terms of cell localization, NOD1 and NOD2 are very differently expressed in cells and tissues throughout the body. NOD1 is extensively expressed in a variety of cell types, whereas NOD2 has been found mostly in professional immune cells (macrophages,^25^ dendritic cells,^26^ and Paneth cells^27^), osteoblasts,^28^ keratinocytes,^29^ intestinal stem cells,^30^ and various epithelial cells.^31^, ^32^, ^33^

### 2.2 Structure

NOD1 and NOD2 are multiple domain proteins consisting of a C‐terminal, leucine‐rich repeat domain (LRR) (also widely accepted as the bona fide sensor domain that is responsible for recognition of ligands), a centrally located nucleotide‐binding oligomerization domain (NACHT) that mediates self‐oligomerization and is crucial for NOD activation, and one (NOD1) or two (NOD2) N‐terminal caspase recruitment domains (CARDs) that interact with downstream signaling molecules.^34^, ^35^ Normally, NODs are kept in a monomeric autoinhabitable state in the cell cytosol, being activated following ligand binding.^19^ In addition to being able to recognize ligands in the cytosol, NODs are capable of trafficking dynamically to the cell membrane and of recognizing bacteria at the point of entry.^36^

### 2.3 Signaling

#### 2.3.1 The canonical signaling pathway (NF‐κB and MAPK)

**Figure 2:**
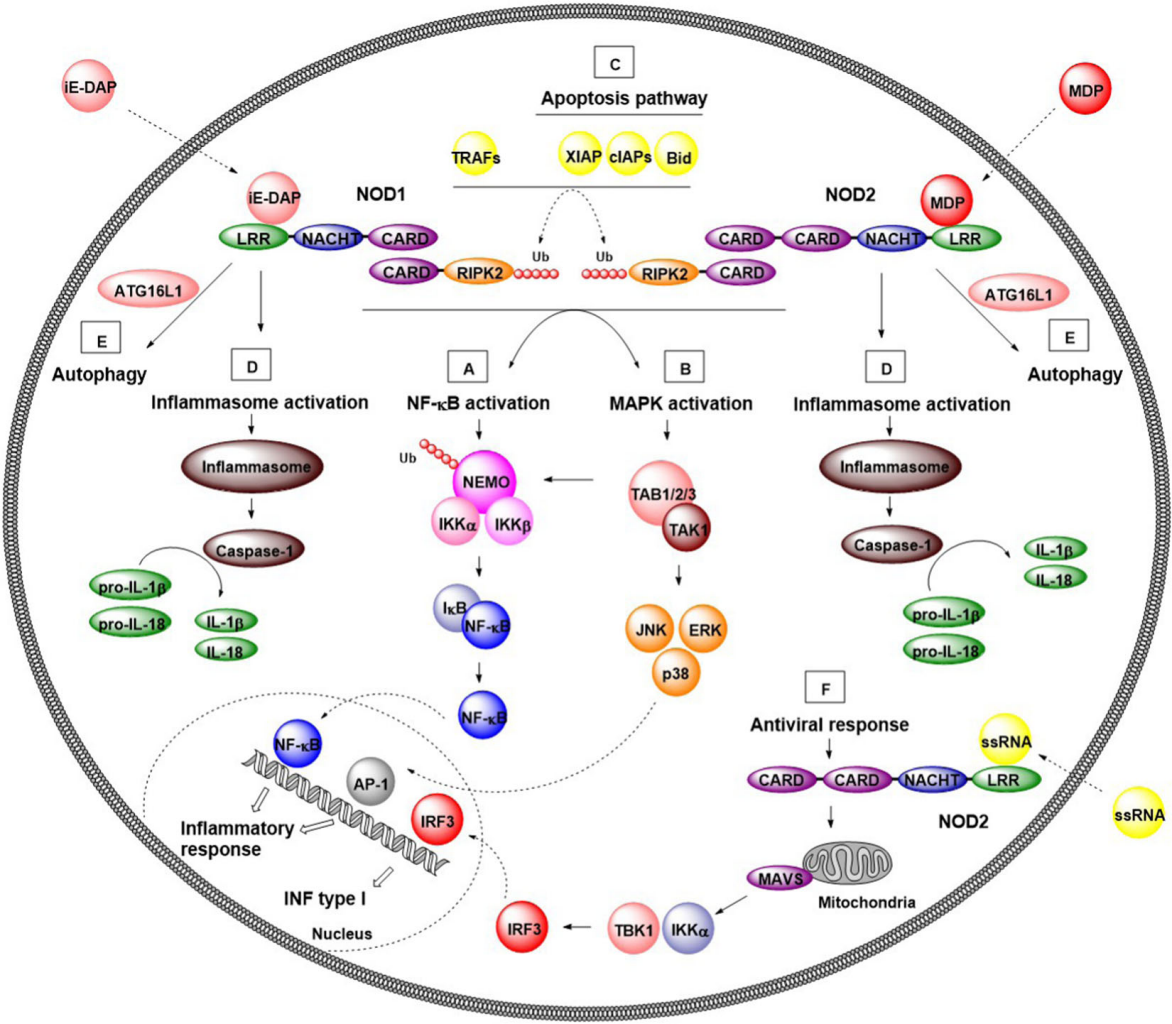
Canonical and noncanonical signaling pathways of NOD1 and NOD2. A, B, NF‐κB and MAPK signaling pathways. NOD1 and NOD2 recognize bacterial PGN fragments, iE‐DAP and MDP, respectively. After ligand recognition, NODs undergo conformational changes and self‐oligomerization through homophilic CARD‐CARD interactions, allowing the recruitment and activation of the adaptor protein RIPK2. In an established protein complex, RIPK2 is polyubquitinated by TRAFs and cIAPs, allowing the recruitment of polyubiquitinated NEMO or TAK1 to the established protein complex. On one hand, NEMO triggers activation of the NF‐κB pathway by phosphorylation of IκB resulting in release NF‐κB transcription factor. The latter translocates to the nucleus where causes induction of pro‐inflammatory genes. On the other hand, TAK1 recruits TAB1/2/3 activating both NF‐κB and MAPK pathway (JNK, ERK, and p38). Activated MAPKs translocate to the nucleus and activate AP‐1 transcription factor resulting transcription of genes involved in inflammatory response. C, Apoptosis pathway. NODs have been reported to interact with the apoptotic pathway indirectly through IAP family of proteins (cIAP1, cIAP2, and XIAP) and proapoptotic protein Bid. D, Inflammasome activation. NODs associate with several other NLRs (NLRP1 and NLRP3) to form inflammasome protein complexes. After detection of their cognate ligand, NLRs interact with ASC protein and procaspase‐1, resulting in formation of inflammasomes. Once formed, inflammasomes activate procaspase‐1, which in turn proteolytically processes pro‐inflammatory cytokines IL‐1β and IL‐18. E, Autophagy. Activated NODs recruit the autophagy protein ATG16L1 to the cell membrane and facilitate the formation of autophagosome around invading bacteria. F, Antiviral response. Viral ssRNA activates NOD2, which translocates to the mitochondria and supposedly binds to MAVS. This promotes formation of a complex with TBK1 and IKKα, which enables activation of interferon regulatory factor (IRF) 3 resulting in production of IFN type I. ASC, apoptosis‐associated speck‐like protein containing caspase recruitment domain; CARD, caspase recruitment domain; cIAP, cellular inhibitor of apoptosis protein; ERK, extracellular signal‐regulated kinase; IAP, inhibitor of the apoptosis; iE‐DAP, d‐glutamyl‐*meso*‐diaminopimelic acid; IFN, interferon; IKK, IκB kinase; IκB, protein inhibitor of NF‐κB; IL, interleukin; JNK, c‐Jun N‐terminal kinase; NOD, nucleotide‐binding oligomerization domain; NF‐κB, nuclear factor κB; MAPK, mitogen‐associated protein kinase; MAVS, mitochondrial antiviral signaling; MDP, muramyl dipeptide; NEMO, nuclear factor κB essential modulator; NLRs, nucleotide‐binding oligomerization domain‐like receptors; PGN, peptidoglycan; RIPK2, receptor‐interacting serine/threonine‐protein kinase 2; TAB, transforming growth factor binding protein; TAK1, transforming growth factor β‐activated kinase 1; TBK1, TRAF‐associated nuclear factor‐κB activator–binding kinase 1; TRAFs, tumor necrosis factor receptor–associated factors; XIAP, X‐linked inhibitor of apoptosis protein [Color figure can be viewed at wileyonlinelibrary.com]

On activation by their native ligands, NODs undergo conformational changes and self‐oligomerization through homophilic CARD‐CARD interactions, allowing the recruitment and activation of the CARD‐containing adaptor receptor‐interacting serine/threonine‐protein kinase 2 (RIPK2). The latter is important for downstream signal transduction.^37^, ^38^, ^39^ In an established protein complex, RIPK2 is later polyubiquitinated by several E3 ubiquitin ligases, namely tumor necrosis factor receptor–associated factors (TRAFs), cellular inhibitor of apoptosis protein (cIAP) 1, cIAP2, and X‐linked inhibitor of apoptosis protein (XIAP).^40^, ^41^, ^42^, ^43^, ^44^, ^45^ Polyubiquitin chains attached to RIPK2 then facilitate the formation and activation of a protein complex consisting of tumor growth factor β‐activated kinase 1 (TAK1) and TAK1‐binding proteins (TAB) 1 to 3.^46^, ^47^ TAK1 is an upstream activator of the inhibitory κB kinase (IKK) complex.^48^ This activation leads to the phosphorylation and degradation of a protein inhibitor of NF‐κB (IκB), resulting in translocation of NF‐κB to the nucleus and transcription of NF‐κB target genes.^42^, ^46^, ^49^ On the other hand, TAK1 also activates three mitogen‐activated protein kinases (MAPKs), namely p38, extracellular signal‐regulated kinase (ERK), and c‐Jun N‐terminal kinase (JNK), resulting in the activation of activator protein 1 (AP‐1) transcription factor.^50^ Moreover, RIPK2 also interacts with the IKKγ/NEMO subunit of IKK complex, resulting in ubiquitination of IKKγ/NEMO and activation of the IKK complex, which is important for downstream NF‐κB activation (Figure 2).^15^

In addition to NF‐κB and MAPK signaling pathways, NODs are involved in the activation of other innate immunity systems such as autophagy, apoptosis, inflammasome activation, and even antiviral response, as described briefly below.

#### 2.3.2 Noncanonical signaling pathways

##### Apoptosis pathway

NODs have been reported to interact indirectly with the apoptotic pathway through the inhibitor of the apoptosis (IAP) family of proteins (cIAP1, cIAP2, and XIAP) as with the proapoptotic protein BH3‐interacting domain death agonist (Bid) (Figure 2).^40^, ^43^, ^51^, ^52^ IAP proteins participate in NOD signaling by polyubiquitinating RIPK2 and consequently stimulating NF‐κB activation and stress kinases activities,^40^, ^43^, ^51^ while Bid has been suggested to bridge the NODs to the IKK complex thereby impacting NF‐κB and ERK activation.^52^ Furthermore, it has been demonstrated that stimulation of NOD1 activates caspase 8, which has been linked to its underlying antitumor activity.^53^, ^54^

##### Autophagy

Autophagy is a highly conserved degradation process in eukaryotic cells, vitally involved in the normal functioning of the innate immune system.^55^, ^56^, ^57^, ^58^ It has recently been discovered that NOD‐mediated recognition of bacteria induces autophagy and bacterial clearance.^59^, ^60^, ^61^ NODs have been shown to recruit the autophagy protein ATG16L1 to the cell membrane, to target bacteria at the point of entry, independently of RIPK2 (Figure 2).^61^ In addition to their role in sensing bacteria, NODs are involved in autophagosome formation. It has been demonstrated that autophagosome formation is induced in epithelial cells, fibroblasts or dendritic cells (DCs) on stimulation by NOD agonists.^59^, ^60^, ^61^ In contrast to the cell membrane targeting function, the induction of autophagy by NOD2 is an RIPK2‐dependent process leading to downstream ERK and p38 activation.^60^ It should be noted that signaling through RIPK2 deactivates protein phosphatase 2A, which negatively regulates NOD‐dependent autophagy.^60^

##### Inflammasome activation

NODs associate with several other NLRs, such as NLRP1 and NLRP3 to form inflammasome protein complexes.^62^, ^63^, ^64^ Inflammasomes are intracellular multiprotein complexes that detect pathogens as well as various sterile stressors including self‐derived DAMPs, alum, asbestos, silica, alloy particles, UV radiation, and skin irritants.^10^ After detection of PAMPs or DAMPs, NLRs interact with adaptor protein ASC (apoptosis‐associated speck‐like protein containing a CARD) and procaspase‐1, resulting in the formation of multiprotein inflammasome complexes. Once formed, inflammasomes activate procaspase‐1, which in turn proteolytically processes pro‐inflammatory cytokines interleukin (IL)‐1β and IL‐18 (Figure 2).^65^

##### Induction of an antiviral response

In addition to the previously described signaling pathways, studies have also provided evidence for PGN‐independent role of NODs.^66^, ^67^ Specifically, NOD2 can act as a cytoplasmic viral PRR that activates an antiviral response, resulting in type I interferon (IFN) production. After detection of viral ssRNA, NOD2 translocates to the mitochondria where it supposedly interacts with mitochondrial antiviral signaling (MAVS) protein. This promotes the formation of a complex with serine/threonine‐protein kinases TBK1 and IKKα, enabling activation interferon regulatory factor (IRF) 3 and resulting in the production of IFN‐β (Figure 2).^15^, ^67^

## 3 THE ROLE OF NOD PROTEINS IN CANCER DEVELOPMENT

Although NODs were initially recognized as receptors for pathogen recognition within the scope of the innate immune response, recent findings have further confirmed their involvement in mechanisms underlying cancer development. On the one hand, NOD activation can prevent, inhibit, or block carcinogenesis by controlling epithelial cell regeneration while, on the other hand, it can promote carcinogenesis via the production of pro‐inflammatory cytokines that contribute to chronic inflammation.^21^, ^68^ Furthermore, increased cancer risk is also associated with the presence of polymorphisms in genes *CARD4* and *CARD15*.^21^ These polymorphisms can produce altered NODs with disrupted cytokine‐producing profiles and therefore pose an increased risk, causing inflammation and cancer. Briefly, NOD2 gene polymorphisms have been associated with increased risk of lymphoma, colorectal, gastric, breast, ovarian, lung, and laryngeal cancers while NOD1 gene polymorphisms have been linked to increased risk of lymphoma, gastric, colorectal, ovarian, prostate, and lung cancer, as well as the cancer types whose etiology is related to Crohn's disease and sarcoidosis.^21^ NODs have been studied to a greater extent in cancers of the gastrointestinal tract, such as colorectal cancer (CRC) and gastric cancer (GC), although studies in breast cancer, oral squamous cell carcinoma (OSCC), head and neck squamous cell carcinoma (HNSCC), and pancreatic cancer (PC) have also been conducted (Table 1). Stimulation of NOD1 and NOD2 was found to be protective in inflammation‐induced CRC,^69^, ^70^, ^71^, ^80^ whereas there was no straightforward answer as to whether activation of NOD1 in the stomach promotes or prevents the development of GC.^72^, ^73^, ^74^ Moreover, NOD1 was found to be upregulated in PC,^79^ HNSCC,^77^, ^78^ OSCC,^76^ and GC,^73^, ^74^ as opposed to certain studies that reported NOD1 downregulation in the cases of OSCC^75^ and GC.^72^ NOD2 was also found to be upregulated in GCs.^74^

**Table 1:** Role of NOD proteins and their expression level in different types of cancer

		Role of NOD proteins and their expression level		
Cancer	NOD protein	Protective (P)/ detrimental (D)		References
Colorectal	NOD1	P	NOD1 deficiency alone or together with a mutation in Apc (ApcMin/+) leads to increased risk of tumor formation in the AOM/DSS mouse model of colon cancer. Increased tumor formation is a consequence of increased intestinal epithelial apoptosis as well as intestinal permeability associated with enhanced inflammatory cytokine production and epithelial cell proliferation.	69
	NOD1	P	NOD1 deficiency in T cells increases risk of tumor formation in mice using AOM/DSS model of colon cancer. NOD1 deficiency in T cells is associated with impaired IFN‐γ production and STAT1 activation.	70
	NOD2	P	NOD2 (or RIPK2) deficiency results in increased susceptibility to tumor formation in AOM/DSS mouse model of colon cancer. Absence of NOD2 (or RIPK2) promotes pro‐inflammatory microenvironment in the intestines leading to enhanced epithelial dysplasia following chemically induced injury.	71
Gastric	NOD1	P	Decreased expression level of NOD1 in *Helicobacter pylori*‐positive GC patients. Stimulation of NOD1 by C12‐iE‐DAP before infection with *Helicobacter pylori* reduced risk of GC development in gerbils.	72
	NOD1/NOD2	D	Increased expression level of NOD1 in *Helicobacter pylori*‐positive and *Helicobacter pylori*‐negative GC patients. Increased expression level of NOD2 in *Helicobacter pylori*‐positive GC patients.	73, 74
Breast	NOD1	P	NOD1 deficiency leads to increased tumor growth in mouse model of breast cancer. Stimulation of NOD1 overexpressed breast cancer cells results in caspase 8–mediated apoptosis.	53, 54
Oral squamous cell carcinoma (OSCC)	NOD1	P	Decreased expression level of NOD1 in OSCC patients. NOD1 expression decreases along with OSCC progression.	75
	NOD1/NOD2	D	NOD1 and NOD2 are apparently expressed in YD‐10B and FaDu cell line. Stimulation of NOD1 and NOD2 in YD‐10B cells by Tri‐DAP and MDP, respectively, results in production of IL‐8 and MAPK activation. Stimulation of YD‐10B cells by MDP results in inhibition of the proliferation and induction of apoptosis.	76
Head and neck squamous cell carcinoma (HNSCC)	NOD1	D	Increased expression level of NOD1 in tumor biopsies, Detroit‐562 and FaDu cell line. Stimulation of NOD1 in HNSCC cells by iE‐DAP increases the production of β‐defensin 2, GM‐CSF, G‐CSF, and upregulates ICAM‐1. NOD1 activation by iE‐DAP increases the apoptosis and decreases the number of dead Detroit‐562 cells.	77
	NOD1	D	Increased expression level of NOD1 (as well as IL‐8 and RIPK2) in tumor biopsies. IL‐8 is a key factor in NOD1‐mediated RIPK2 activation and HNSCC progression.	78
Pancreatic	NOD1	D	Increased expression level of NOD1 in peripheral blood leukocytes of pancreatic cancer.	79

Abbreviations: AOM, azoxymethane; CSF, colony‐stimulating factor; DSS, dextran sulfate sodium; GC, gastric cancer; ICAM‐1, intercellular adhesion molecule‐1; iE‐DAP, d‐glutamyl‐*meso*‐diaminopimelic acid; IFN, interferon; IL, interleukin; MDP, muramyl dipeptide; NOD, nucleotide‐binding oligomerization domain; RIPK2, receptor‐interacting serine/threonine‐protein kinase 2; STAT1, signal transducer and activator of transcription 1; Tri‐DAP, l‐alanyl‐γ‐d‐glutamyl‐*meso*‐DAP.

## 4 TARGETING NOD RECEPTORS IN CANCER IMMUNOTHERAPY

Extensive research has shown that cancer is not just a group of malignant cells but a complex structure within a tumor microenvironment (TME).^81^, ^82^, ^83^ Besides malignant cells, TMEs comprise a variety of immune and nonimmune cell types that, in concert with the many other factors that they secrete, create an effective environment that favors tumor growth and metastatic dissemination.^8^, ^81^ Infiltration of TME by immune cells such as macrophages, lymphocytes, natural killer (NK) cells, and DCs in the early stages of tumor development is crucial for an appropriate anticancer immune response. Unfortunately, the beneficial effect produced by these cells is often inhibited by the action of immunosuppressive cells, including regulatory T cells, type 2 (M2) macrophages, and myeloid‐derived suppressor cells (MDSCs) that also infiltrate the TME of developing tumors.^83^ In such an immunosuppressive environment, cancer cells are able to adapt and remain undetected by host immunosurveillance.^83^ The overarching goal of cancer immunotherapy is to overcome the immunosuppression in TME, thereby enabling immune cells to effectively eliminate cancer cells without causing intolerable side‐effects.^84^ To achieve this, various strategies have been used, among which targeting of PRR, including NOD1 and NOD2, constitutes an interesting and novel approach that could be used as an adjunct to current cancer therapies.^8^ In terms of anticancer activity, NOD agonists can act as (i) immunotherapeutics or (ii) adjuvants in cancer vaccines whereas NOD antagonists have recently proposed to mediate their antitumor activity by preventing the formation of an inflammatory TME.

### 4.1 NOD agonists as immunotherapeutic agents

When NOD agonists act as immunotherapeutics, they activate the cytotoxic potential of immune cells residing in the TME and, consequently, facilitate their engagement with cancer cells. Such enhancement of anticancer immunity has been investigated in the context of NOD2 agonists which, mainly, activate monocytes and macrophages^85^ although stimulation of NK cells^86^, ^87^ and DCs^88^, ^89^, ^90^ has also been reported. In general, two possible mechanisms on how NOD agonists activate the antitumor activity of macrophages have been suggested. They can either induce tumoricidal macrophages that, in turn, attack cancer cells or stimulate macrophages to mediate anticancer activity indirectly by the release of pro‐inflammatory molecules and other factors. Moreover, macrophages also collaborate with Th1 cells to effectively recognize and eliminate malignant cells. Specifically, type 1 (M1) macrophages and Th1 cells reinforce one another, with M1‐produced IL‐12 maintaining the Th1 phenotype and Th1‐produced IFN‐γ maintaining the M1 phenotype.^91^ In this Th1‐driven environment, pro‐inflammatory cytokines such as tumor necrosis factor (TNF)‐α, IL‐1, and IL‐6 are also involved in cancer elimination by stimulating various aspects of antitumor immunity, including recruitment of macrophages and T cells from the circulation and stimulation of leukocyte tumoricidal functions.^91^

### 4.2 NOD agonists as adjuvants for cancer vaccines

NOD agonists have a potential for use as adjuvants in cancer vaccines and therefore enhance innate as well as adaptive immune responses toward coadministered antigens with insufficient immunostimulatory capabilities.^84^, ^92^, ^93^, ^94^ In fact, NOD ligands have been involved in vaccination strategies throughout the 20th century, being essential components of Freund's Complete Adjuvant (FCA), one of the most potent and widely used adjuvants in animals. Among them, DAP‐containing peptides have demonstrated favorable adjuvant activity, whereas MDP was recognized to be the minimal structure required for the adjuvanticity of FCA.^95^, ^96^, ^97^ MDP and other muropeptides exert their immune‐enhancing effects through several mechanisms. For example, they increase expression of cell surface markers, which are involved in cell adhesion and presentation of antigens, thereby stimulating phagocytic and antimicrobial activity as well as increasing antibody‐mediated cytotoxicity.^98^ Moreover, MDP is reported to increase immune responses of other immunomodulatory molecules such as IFN‐γ and to synergize with several cytokines, thereby stimulating differentiation and proliferation of lymphocytes.^99^, ^100^

## 5 NOD LIGANDS AS ANTICANCER AGENTS

### 5.1 NOD1 agonists

**Figure 3:**
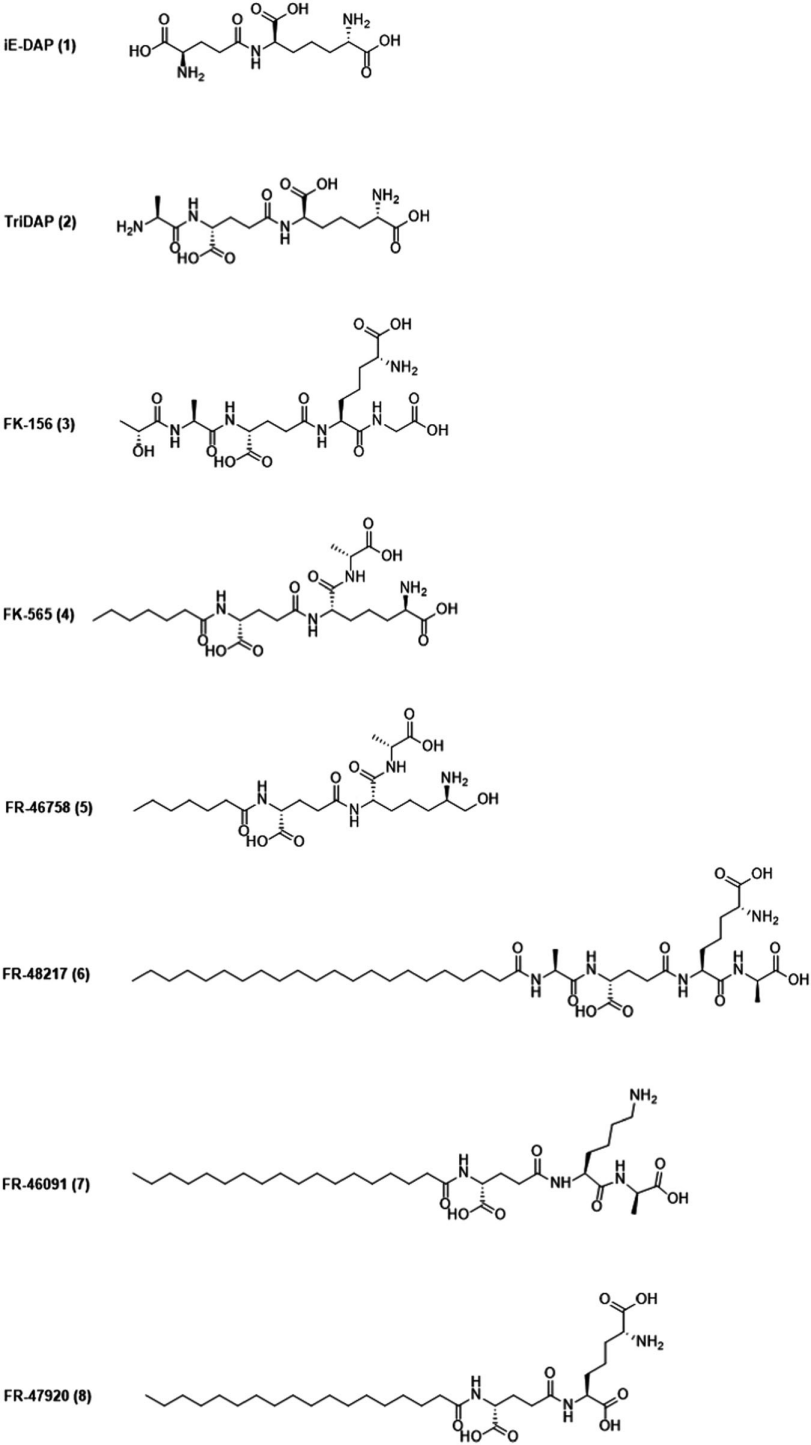
Chemical structures of iE‐DAP, Tri‐DAP, and NOD1 agonists with anticancer activity. iE‐DAP, d‐glutamyl‐*meso*‐diaminopimelic acid; NOD, nucleotide‐binding oligomerization domain; Tri‐DAP, l‐alanyl‐γ‐d‐glutamyl‐*meso*‐DAP

NOD1 senses different DAP‐containing ligands that originate from bacterial PGN. Among them iE‐DAP (**1**; Figure 3) is recognized as the minimal component sufficient for NOD1 activation. Elongation by an additional alanine resulted in Tri‐DAP (**2**), which turned out to be an even more potent NOD1 agonist.^11^, ^34^, ^101^ In addition to these DAP‐containing NOD1 agonists released from bacteria, various iE‐DAP analogs with immunostimulatory activities have been designed and synthesized, mostly by introducing lipophilic moieties to the d‐glutamyl (d‐Glu) portion of the iE‐DAP molecule.^102^ For example, Jakopin et al^103^ synthesized several iE‐DAP analogs with lauroyl and didodecyl moieties attached to the amino group of the d‐Glu residue. These NOD1 agonists alone (10 µM) or in synergy with lipopolysaccharide (LPS) (1 ng/mL) exhibited significant immunostimulatory effects in human peripheral blood mononuclear cells (PBMCs) resulting in increased cytokine production (TNF‐α, IL‐6, IL‐8, and IL‐10).

In addition to immunostimulation, there is evidence suggesting that NOD1 agonists also possess anticancer activity.^104^, ^105^, ^106^ In the 1980s, researchers from Fujisawa Pharmaceutical Co synthesized a series of structurally related *meso*‐DAP incorporating analogs that exhibited antitumor activity in vivo.^104^ For the first screening of compounds, two intratumoral injections of *meso*‐DAP analog (10‐100 µg/site) were administered in DBA/2 mice with established P388 solid tumors. Among 21 synthetic analogs tested, only six compounds, namely FK‐156 (**3**), FK‐565 (**4**), FR‐46758 (**5**), FR‐48217 (**6**), FR‐46091 (**7**), and FR‐47920 (**8**) showed promising tumor growth inhibition (20%‐50%). Moreover, significant tumor growth inhibition (20%‐40%) was also observed when P388 tumor‐bearing mice received two subcutaneous injections of **3**, **4**, **5**, and **6** (6 mg/kg) or multiple systemic injections of **4** (200 µg/kg). In contrast to the substantial antitumor activity of **3**, **4**, and **5** observed in vivo, these three compounds at 1 mg/mL concentration demonstrated no toxicity against P388 cells in vitro suggesting that these compounds boost the antitumor activity of immune effector cells such as monocytes, macrophages, or NK cells.^104^ In subsequent studies, **4** was indeed shown to stimulate NK cells and induced tumoricidal activities of murine macrophages.^105^, ^106^ Remarkably, small amounts of **4** (more than 0.1 µg/kg) stimulated NK cell activity and inhibited experimental lung metastasis formation when administered prophylactically 2 or 3 days before inoculation of B16 melanoma cells.^106^ Conversely, in the study of Inamura et al^106^ doses as high as 100 µg/kg of **4** were not effective in inhibiting the formation of lung metastasis administered 3 days after B16 tumor cell inoculation. Since NK cells were not effective in controlling pulmonary metastasis when administered 3 days after B16 tumor cell inoculation, they probably destroy only circulating but not extravascular, metastatic cells. Interestingly, repeated intravenous or subcutaneous injections of **4** given at high doses of 1 to 10 mg/kg after B16 tumor cell inoculation significant reduced the number of pulmonary metastases in an established experimental lung metastasis model.^106^ In our opinion, the difference between the efficacy of prophylactic and therapeutic treatment is probably due to the different mechanisms responsible for the antimetastatic activity. Namely, it is likely that antimetastatic effect observed in the case of therapeutic treatment involves macrophage activation, and not NK cell activation since repeated intraperitoneal injections of **4** at high doses (more than 10 mg/kg) significantly increased the cytotoxicity of murine peritoneal macrophages. Similarly, Schultz et al^105^ also confirmed the role of macrophages as the primary effectors of **4**‐induced antimetastatic activity. Specifically, reduced M109 lung metastasis formation was observed when high doses of **4** (1‐10 mg/kg) were administered prophylactically 2 to 4 days before the inoculation of M109 tumor cells in an experimental metastasis model. This protective activity was, however, abolished by the selective macrophage inhibitor 2‐chloroadenosine, thus further corroborating the notion that macrophage activation is underlying the antimetastatic activity of **4** in this experimental model.^105^ In spite of the fact that activation of macrophages was proposed to be the primary mechanism of **4**‐mediated prophylactic activity against M109 metastasis, further studies are needed to determine if other effector mechanisms, such as NK cell activation, are also involved in the observed antimetastatic activity. Although the exact mechanism of action has not been fully characterized, NOD1 agonists turned out to be effective immunomodulators with promising antimetastatic activity when used as prophylactic treatment. Among all described compounds, **4** efficiently inhibited th growth of lung metastases in vivo while exhibiting minimal toxicity in animal studies, and thus emerged as a prospective immunotherapeutic agent. In fact, compound **4** indeed entered phase I clinical trial for use in cancer therapy but unfortunately, this study was discontinued. Nevertheless, from our perspective, the field of NOD1 agonists still holds a lot of potential. For example, several recently synthesized potent NOD1 agonists^102^, ^103^ have yet to be examined for their anticancer activity.

### 5.2 NOD2 agonists

**Figure 4:**
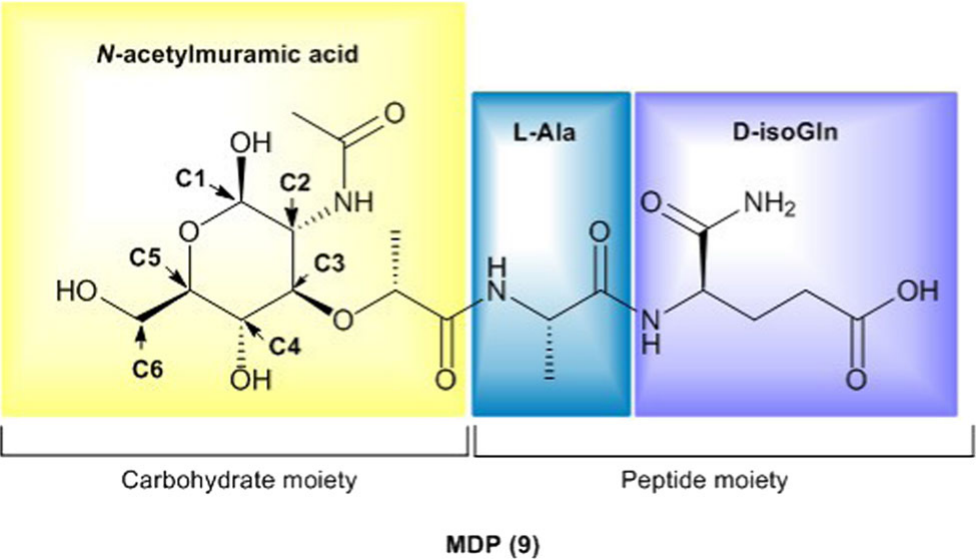
Chemical structure of muramyl dipeptide (MDP). MDP molecule is composed of a carbohydrate moiety represented by *N*‐acetylmuramic acid, and peptide part consisting of l‐Ala and d‐isoGln. Positions of C atoms at the carbohydrate moiety, which are important for further modifications are marked with arrows [Color figure can be viewed at wileyonlinelibrary.com]

MDP (**9**) is the minimal structural component of PGN that activates NOD2. Structurally speaking, MDP is a small molecule composed of an *N*‐acetylmuramic acid linked to a dipeptide consisting of l‐alanine (l‐Ala) and d‐isoglutamine (d‐isoGln) (Figure 4). Due to its low molecular weight, MDP is highly water soluble and rapidly excreted from the body,^107^ resulting in weaker in vivo activity.^108^ To overcome this issue and obtain NOD2 agonists with improved immunostimulatory and anticancer activities, the parent structure of MDP has been (i) incorporated into different nanocarrier delivery systems such as liposomes and nanocapsules or (ii) equipped with various classes of compounds including lipophilic molecules, biomolecules, drugs, and many others. To date, several hundred MDP analogs have been synthesized (reviewed in^109^, ^110^). According to the type of modifications in MDP, NOD2 agonists possessing anticancer activities can be classified into four groups namely, lipophilic derivatives, hydrophilic derivatives, conjugates, and desmuramylpeptides.

#### 5.2.1 Lipophilic MDP derivatives

As noted previously, lipophilic derivatives with improved immunostimulatory and anticancer activities have been designed to overcome their rapid elimination and consequently weaker stimulation of immune cells with MDP in vivo. To achieve this goal, lipophilic moieties were incorporated into MDP, either at the muramyl moiety at positions C1, C6, or at the C‐terminus of the peptide part. Encapsulation of lipophilic MDP derivatives into liposomes showed even better results in terms of effective immune stimulation since these derivatives are more readily incorporated into and retained within liposomes than MDP, thus leading to increased efficacy. The identification of the optimal liposome‐encapsulated formulations is based on numerous preclinical and animal studies and ensures effective drug delivery into different parts of the body. Such liposome formulations allow for enhanced uptake by monocytes and macrophages, the primary targets of NOD2 agonists via phagocytosis, in turn, they are concentrated in the lysosomal compartment where they are degraded, thus finally releasing lipophilic MDP derivatives.

The group of lipophilic MDP derivatives comprises a variety of molecules such as 6‐*O*‐acyl derivatives of MDP with mycolic, hydroxyl fatty and quinonylalkanoic acids as well as stearoyl, glycoside, glycerol dipalmitate (GDP), and phosphatidylethanolamine derivatives of MDP. Mifamurtide is certainly the most important representative of this group. Due to its favorable pharmacokinetics, pharmacodynamics, and clinical efficacy, it has already been granted a marketing authorization in Europe for use in combination with chemotherapy in patients with high‐grade osteosarcoma after complete surgical removal of the primary tumor.

##### Mifamurtide

**Figure 5:**
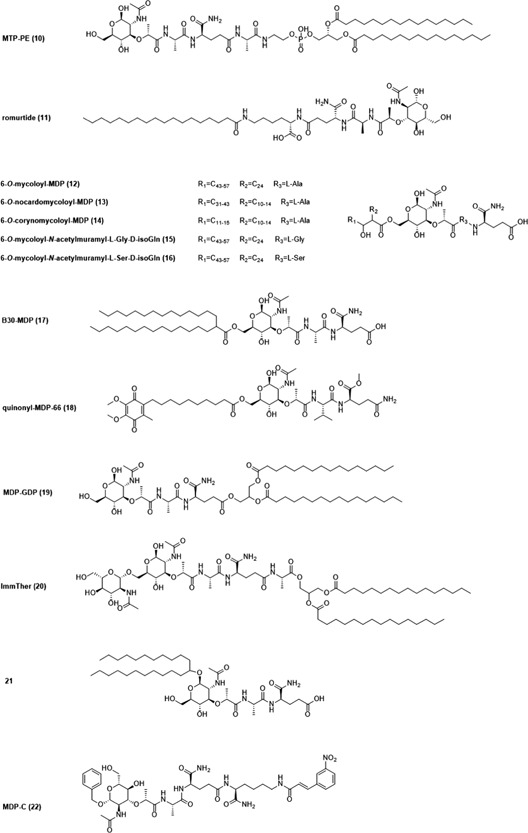
Lipophilic muramyl dipeptide (MDP) derivatives with anticancer activity

Muramyl tripeptide phosphatidylethanolamine or MTP‐PE (**10**; Figure 5) is a fully synthetic lipophilic derivative of MDP that has monocyte‐ and macrophage‐activating properties similar to those of the parent compound, with additional improvements in terms of longer half‐life in plasma and lower toxicity.^108^ In mifamurtide, **10** is encapsulated into multilamellar liposomes by combining the active substance with phospholipids at a ratio 1:250. This formulation facilitated the delivery of **10** to monocytes and macrophages, especially those in the liver, lungs, and spleen. Following phagocytosis by monocytes and macrophages, liposomes incorporating **10** are degraded and release **10**, resulting in activated monocytes and macrophages.^111^ It has been proposed that the anticancer activity of **10** is associated with its ability to induce tumoricidal monocytes and macrophages that attack cancer cells directly, as well as with the release of pro‐inflammatory molecules such as TNF‐α, IL‐1, IL‐6, IL‐8, and IL‐12.^112^, ^113^, ^114^, ^115^ In vitro studies showed that human monocytes activated by mifamurtide selectively recognized and killed tumor cells, while no cytotoxic effect toward normal cells was demonstrated.^113^, ^116^, ^117^, ^118^, ^119^ This selective cytotoxicity was observed even under cocultivation conditions of the tumor and normal cells.^113^ Moreover, mifamurtide also synergized with IFN‐γ to increase the tumoricidal activity of human monocytes.^117^, ^118^, ^120^ For example, Sone et al^120^ demonstrated that monocytes derived from healthy donors exhibited cytotoxicity against human A375 melanoma cells when incubated in vitro with mifamurtide (500 nM) or IFN‐γ (100 U/mL). Interestingly, the cytotoxic effect against A375 tumor cells was significantly enhanced when monocytes were treated with low doses of mifamurtide (50 nM) and IFN‐γ (10 U/mL), indicating that mifamurtide and IFN‐γ–activated monocytes in a synergistic manner. From our point of view, synergistic actions of NOD2 agonists with cytokines are of particular importance, given that the doses used can be drastically reduced resulting in no or fewer undesirable side effects. Furthermore, studies in dogs revealed a beneficial role of mifamurtide in the treatment of spontaneous osteosarcoma, which has many similarities with osteosarcoma in humans. In both, dogs and humans, osteosarcoma arises from long bones and has the same pattern of metastasis, with more than 80% of metastases occurring in the lungs. Specifically, mifamurtide (2 mg/m^2^, dosed twice weekly for 8 weeks) significantly improved overall survival as compared with placebo in dogs with spontaneous osteosarcoma and splenic hemangiosarcoma when used as part of adjuvant therapy after resection of primary tumor.^121^, ^122^, ^123^, ^124^ In contrast, mifamurtide was not effective in mice with high tumor burden, or in cats and dogs with mammary metastatic tumors, which suggests that the impact of macrophage activation on controlling tumor growth depends on tumor burden and tumor location.^125^, ^126^, ^127^ Moreover, mifamurtide also demonstrated no strong interactions in terms of enhancing macrophage activation with chemotherapeutics such as doxorubicin (DOX), cisplatin (CDDP), methotrexate (MTX), and ifosfamide (IFO), which are usually used concomitantly with mifamurtide in therapy of osteosarcoma.^128^, ^129^, ^130^ Briefly, Kleinerman et al^130^ assessed the tumoricidal activity of blood monocytes isolated from osteosarcoma patients receiving CDDP, high‐dose MTX, cyclophosphamide (CTX), or DOX following in vitro activation with mifamurtide (liposomes containing 100 nmol **10**) and observed no difference when compared to monocytes isolated from normal donors. Of note, **10** was also studied as an adjuvant in cancer vaccines.^131^ The study of Bergers et al^131^ demonstrated that **10** in combination with tumor antigens effectively induced specific protective antitumor immunity against SL2 lymphosarcoma cells. The studied mice received two subcutaneous immunizations (at a 10‐day interval) using liposomal formulations containing tumor antigens (solubilized from crude membranes of SL2 cells) and different immunomodulators (**10**, lipid A, dimethyl dioctadecyl ethanolamine). Ten days after the second immunization mice were challenged with live SL2 cells. Mice immunized with liposomes containing tumor antigens and **10** (20 µg/dose) provided better protection against the challenge with SL2 cells in comparison with alternative immunomodulators. On the other hand, mice immunized with tumor antigens derived from unrelated P825 tumor and **10** were not able to reject a challenge with SL2 cells evidently highlighting the need to develop specific antitumor immunity. The obtained results also revealed that mouse peritoneal macrophages isolated from immunized mice 5 to 7 days after tumor challenge demonstrated high nonspecific cytotoxicity in vitro (macrophages destroyed the SL2 as well as the nonrelated P815 cells), and that no major cytotoxic lymphocyte activity or substantial cytotoxic antibody titers were detectable. These results indicate that although tumor cells can be destroyed by nonspecific macrophage cytotoxicity, T cells should be involved at least in the induction of tumor immunity due to the specificity in the tumor rejection. Although exact mechanism underlying inducing antitumor immunity due to the specificity in the tumor rejection by liposomal formulations containing tumor antigens and **10** has not been fully characterized, **10** clearly showed the potential to be used as an adjuvant in cancer vaccines.

The clinical efficacy of mifamurtide has been evaluated in a large number of phase I and II trials.^132^ Additionally, one large, randomized, prospective, open‐label, multicenter phase III trial (Intergroup Study 0133) has been conducted evaluating the addition of mifamurtide to a three‐drug combination (DOX, CDDP, and high‐dose MTX) and to a four‐drug combination (DOX, CDDP, high‐dose MTX, and IFO) chemotherapy for the treatment of osteosarcoma.^132^, ^133^ Briefly, after the resection of the primary tumor, three‐ or four‐drug chemotherapy was given to patients to complete the full course of therapy. Mifamurtide treatment started simultaneously with chemotherapy following surgery. Mifamurtide was administered intravenously at doses 2 mg/m^2^ twice weekly for 12 weeks, and then twice weekly for an additional 24 weeks (altogether 48 doses in 36 weeks). Importantly, the results demonstrated that addition of mifamurtide was associated with a statistically significant improvement in 6‐year overall survival (but not event‐free survival) in patients with newly diagnosed, high‐grade, nonmetastatic, resectable osteosarcoma in comparison to the patients who did not receive mifamurtide treatment (*P* = 0.03).^132^, ^133^ Currently, mifamurtide holds an orphan drug status in the United States and is marketed in Europe for the treatment, in combination with other chemotherapeutics, of high‐grade, nonmetastatic, resectable osteosarcoma in children, adolescents, and young adults (aged between 2 and 30 years), following complete surgical removal. The recommended regimen of mifamurtide is 2 mg/m^2^ intravenously administered over 1 hour twice weekly (at least 3 days apart) for an initial 12 weeks, followed by 2 mg/m^2^ once weekly for additional 24 weeks (it amounts to 48 doses in 36 weeks). In general, mifamurtide therapy is safe and well tolerated. The major adverse events are fever and chills, which are usually transient and associated with initial administration. Most patients rapidly develop tolerance leading to no adverse events with subsequent administration.^115^, ^132^ Although the exact mechanism by which mifamurtide improves overall survival in patients has not been fully elucidated, it may eliminate micrometastasis or tumor cells after surgery that are not removed by or are resistant to, chemotherapy.^115^, ^132^ In our opinion, it may be possible that mifamurtide could exhibit similar beneficial effects in the therapy of other cancers, especially those which predominantly metastasize to the lungs. Mifamurtide should be further examined to ascertain and potentially harness its potential in the treatment of other cancers.

##### Romurtide

Romurtide (**11**), also known under names MDP‐Lys(L18) and muroctasin, is a synthetic stearoyl‐MDP derivative and an effective immunostimulant in vitro and in vivo.^134^, ^135^ When injected subcutaneously for 10 consecutive days into healthy cynomolgus monkeys **11** (1 mg/dose) significantly increased the number of peripheral neutrophils, monocytes, and platelets. This effect may be the consequence of the ability of **11** to augment the production of several cytokines by the monocytes including colony‐stimulating factors (CSFs), IL‐1, and IL‐6, which have central roles in the regulation of hematopoiesis.^136^ Similar results, in terms of increased hematopoiesis, were also obtained in immunosuppressed mice in which multiple injections of **11** (100 µg/dose) effectively restored the white blood cell count, mainly due to an increase in neutrophil counts.^137^ In both studies (healthy cynomolgus monkeys and mice), the increase in white blood count may be attributable to the augmenting effect of **11** on the production of CSFs, followed by the proliferation and differentiation of stem cells in bone marrow.^136^, ^137^ Due to the success in animal studies, **11** was entered into clinical trials in which it demonstrated a restorative effect on leukopenia in cancer patients.^138^, ^139^ In 1991, **11** (trade name Nopia) was put in the market in Japan for treating cancer patients with leukopenia induced by chemotherapy or radiotherapy.^134^, ^135^ Besides its important role in stimulating hematopoiesis and immune functions, **11** also elicits antitumor immunity against tumors and metastases in vivo.^88^, ^140^, ^141^ Yoo et al^141^ investigated the antimetastatic effect of **11** in three highly metastatic cancers in mice, namely B16‐BL6 melanoma, colon 26‐M3.1 carcinoma, and L5178Y‐ML25 T lymphoma. A single subcutaneous injection of **11** (100 µg) given 2 or 4 days before tumor cell inoculation caused a significant reduction of lung metastasis of B16‐BL6 melanoma (60%) and colon 26‐M3.1 carcinoma (25%‐40%) as well as liver metastasis of L5178Y‐ML25 T lymphoma (65%‐70%). Although similar treatment was not effective when **11** was administered 1 or 3 days after tumor cell inoculation, five doses of **11** (100 µg/dose) into B16‐BL6 bearing mice after tumor cell inoculation again achieved a significant reduction of experimental and spontaneous lung metastasis. Given the fact in vitro studies demonstrated that **11** increased tumoricidal activity of mouse peritoneal macrophages against B16‐BL6 and that serum of mice treated with **11** inhibited growth of L929 cell line (TNF‐α sensitive cell line) it was suggested that the antimetastatic activity of **11** was associated with enhanced nonspecific immune responses of hosts, including activation of macrophages and induction of cytotoxic factors such as TNF‐α.^141^ Activation of macrophages by **11** was also proposed as the underlying mechanism of antimetastatic activity in the study of Nitta et al,^140^ in which free and liposome‐encapsulated **11** inhibited, lung metastasis of transplantable osteosarcoma in hamsters when the **11** was given before or after surgical removal of the primary tumor. Specifically, treatment with free **11** given at a dose 50 µg daily or liposomal **11** given at a dose 20 µg twice a week, which started 3 weeks after tumor transplantation and immediately after primary tumor removal and lasted 4 weeks, resulted in inhibition of lung metastasis for 45% and 40%, respectively, when compared to untreated controls. Despite the fact that both free and liposome‐encapsulated **11** effectively eliminated lung metastases, liposome‐encapsulated **11** exhibited a far greater inhibitory effect than free **11** (40 vs. 350 µg/week), apparently due to the longer retention of the liposomal form in the lung.^140^ In contrast to the beneficial activity of **11** in terms of suppressing lung metastasis, it exhibited no significant effect on primary tumor mass. The precise reason for that is not clear, but it may be linked to the accelerated growth of transplanted osteosarcoma with time, thus surpassing the tumoricidal activity of activated macrophages.^140^ Furthermore, the antitumor effect of **11** was also studied in combination with IFN‐β, which has already shown promising results in the treatment of malignant melanoma.^88^ An in vitro study demonstrated that **11**, in combination with IFN‐β, synergistically augmented activation of human monocyte‐derived DCs (MoDCs), resulting in the production of the pro‐inflammatory cytokines TNF‐α, IL‐6, and IL‐12. Moreover, both T cells cocultured with **11** and IFN‐β–treated MoDCs produced significant levels of IFN‐γ, the pivotal cytokine involved in the Th1 response against malignancy. Due to the promising results obtained in vitro, the **11**/IFN‐β combination was further studied in murine models of melanoma. It was demonstrated that five intradermal injections of **11** (100 ng‐1 µg) and IFN‐β (10,000 U), given 8 to 10 days after B16‐F10 melanoma tumor cell injection significantly augmented the antitumor effect of IFN‐β in a dose‐dependent manner.^88^ It should be noted that doses as **11** as low as 100 ng were already sufficient for a statistically significant increase of IFN‐β–mediated antitumor activity against mouse melanoma. However, further investigations are needed to determine whether the therapy with a combination of **11** and IFN‐β could be beneficial for melanoma patients.

##### 6‐O‐acyl‐MDP derivatives

Efforts to develop new potent antitumor agents by modifying the MDP molecule led to the discovery of 6‐*O*‐acyl derivatives of MDP in which the muramyl moiety was conjugated at the C6 position with various lipophilic molecules. In particular, conjugations of MDP with mycolic, hydroxyl fatty, and quinonylalkanoic acids showed some promising results. Azuma et al^142^ carried out extensive studies using 6‐*O*‐acyl derivatives of MDP in which the latter was coupled with natural mycolic acid isolated from bacterial cell walls.^143^ In the context of antitumor activity, administration of 100 µg 6‐*O*‐mycoloyl‐MDP (**12**), 6‐*O*‐nocardomycoloyl‐MDP (**13**), 6‐*O*‐corynomycoloyl‐MDP (**14**), or 6‐*O*‐mycoloyl‐*N*‐acetylmuramyl‐l‐Gly‐d‐isoGln (**15**) in an oil‐based vehicle suppressed growth of fibrosarcoma in mice while 6‐*O*‐mycoloyl‐*N*‐acetylmuramyl‐l‐Ser‐d‐isoGln (**16**) was active in terms of regression of an established line 10 hepatoma in guinea pigs.^142^, ^143^ To avoid the ambiguities concerning the question whether a certain structural variation or the heterogeneity in the natural mycolic acid is required for the antitumor activity, new derivatives were designed, in which natural mycolic acid was substituted by pure synthetic fatty acids of high molecular weight.^144^ Among them, compound B30‐MDP (**17**), a mycoloyl‐mimic long‐chain fatty acid derivative of MDP, was recognized as a strong adjuvant capable of inducing antitumor immunity. Kataoka et al^145^, ^146^ used a tumor vaccine composed of X‐ray–irradiated line 10 hepatoma cells or acute B cell leukemia cells EN‐L2C in combination with small amounts of **17** (5 µg/dose) to protect guinea pigs against hepatocarcinoma or acute B cell leukemia. Moreover, a similar tumor vaccine formulation also enhanced the activity of cytotoxic killer T cells to inhibit liver metastases of L5178Y‐ML25 lymphoma cells in mice when X‐ray–irradiated L5178Y‐ML25 lymphoma cells and **17** (100 µg/dose) were injected before or after tumor inoculation.^147^ Since MDP derivatives coupled with mycolic and synthetic long chain fatty acids showed promising antitumor activity, the MDP molecule was further modified by introducing the quinonyl 10‐(2,3‐dimethoxy‐5‐methyl‐1,4‐benzoquinon‐6‐yl)decanoic acid, a highly lipophilic analog of ubiquinones, which resulted in the identification of quinonyl‐MDP‐66 (**18**).^148^ In vivo studies demonstrated that a single intradermal injection of **18** (100 µg) administered in a PBS suspension effectively suppressed the growth of Meth A fibrosarcoma in mice.^148^, ^149^, ^150^ In addition, compound **18** was also tested in an oil‐based vehicle following the discovery that oil vehicles including squalene and squalane were required to engage the antitumor activity of some mycobacterial cell‐wall extracts in vivo.^151^ Experiments in strain‐2 guinea pigs with established line‐10 hepatocarcinoma revealed that 4 intratumoral injections of **18** (0.1‐0.4 mg) incorporated into squalene (or squalane) vehicle oil caused a complete tumor regression resulting in 7 tumor‐free animals out of 7. In contrast, multiple injections of **18** (0.4 mg) in PBS or treatment with squalene (or squalene) vehicle oil alone were less effective in terms of tumor regression resulting in only 2 or even 0 to 1 tumor‐free animals out of 7, respectively.^151^ It is worth noting that despite squalene (or squalane) vehicle oil alone showed no significant antitumor activity in that tumor model, several squalene‐based oil‐in‐water emulsion such as FM59 have been recognized as efficient adjuvants and are already used in various vaccines.^84^ Moreover, two injections of **18** (400 µg/dose) also restored the depressed allogeneic cell‐mediated cytotoxicity of spleen cells in 3LL‐bearing mice when administered intraperitoneally, intravenously, or intratumorally.^152^

##### MDP‐GDP

Conjugation of MDP to GDP led to the formation of lipophilic MDP‐GDP (**19**) which was recognized as a very potent immunostimulant, especially when incorporated into liposomes. Specifically, liposomal **19** was able to induce the cytotoxic activity of macrophages in in vitro and in situ studies, as well as antimetastatic activity in vivo.^153^, ^154^, ^155^, ^156^, ^157^, ^158^ Phillips et al^153^ incorporated **19** into liposomes composed of distearoylphosphatidylcholine (DSPC) and phosphatidylserine (PS) (7:3 molar ratio). They discovered that liposomal **19** was far more efficient in inducing alveolar macrophage cytotoxicity than liposomal MDP or free MDP (10‐ and 7000‐fold, respectively). Moreover, these **19**‐containing liposomes efficiently accumulated in the lungs of normal mice and activated murine alveolar macrophages to become cytotoxic against B16‐BL6 tumor cells in vitro. Based on these findings, liposomal **19** was further studied in mice with lung metastases of B16‐BL6 melanoma. Five intravenous injections of liposomal **19** (10 µg) were given to tumor‐bearing mice resulting in significant reduction of lung metastases whereas treatment of tumor‐bearing mice with control liposomes or free MDP (10 µg) had no effect. The similar antimetastatic effect was observed when B16‐BL6 tumor‐bearing mice were treated with liposomes containing **19** or two other MDP‐GDP derivatives, namely GDP derivative of murabutide (hydrophilic, apyrogenic MDP derivative with immunoadjuvant and antitumor activity) and MDP(d,d) (MDP derivative completely devoid of immunoadjuvant activity).^154^ Mice with lung metastases received five intravenous injections of liposomal **19**, murabutide‐GDP, or MDP(d,d)‐GDP resulting in 53%, 36%, and 71% fewer number of lung metastases than control mice, respectively. The order of the ability of liposomal MDP‐GDP derivatives to enhance macrophage activation in vitro and in situ was in fact in good agreement with that observed for the antimetastatic effect. This is particularly interesting since MDP(d,d) itself is completely devoid of immunoadjuvant activity whereas its conjugation with GDP at the terminal amino acid led to a very potent inducer of macrophages and antimetastatic activity. It has been proposed that the difference in the activity is a consequence of its structure. Also MDP(d,d)‐GDP is more resistant to lysosomal enzyme hydrolysis resulting in long‐lived depots within macrophages. In addition to enhancing the cytotoxic potential of alveolar macrophages, **19** incorporated into liposomes also showed promising results in stimulating the tumoricidal activity of macrophages in liver.^155^, ^156^, ^157^, ^158^ To achieve optimal delivery to the liver, **19** was incorporated into liposomes composed of (i) DSPC and PS (7:0.3 molar ratio)^153^, ^155^, ^157^, ^158^ or (ii) DSPC and dimyristoylphosphatidylglycerol (DMPG) (10:1 molar ratio).^156^ Philips et al^158^ demonstrated that liposomal **19** was 16‐fold more effective than liposomal MDP or 2400‐fold more effective than free MDP in inducing Kupffer cell cytotoxic activity in vitro. Moreover, Kupffer cells were also activated after administration of a single intravenous injection of liposomal **19** (0.1‐1 µg/dose) into healthy mice.^158^ More importantly, prophylactic or therapeutic treatment with liposomal **19** (0.1‐1 µg/dose), but not free MDP or control liposomes, resulted in a significant reduction in the number of metastasis in B16‐F10 melanoma–bearing mice. Specifically, prophylactic or therapeutic treatment led to a 70% to 90% reduction of liver tumor burden.^155^ Similar results were obtained by Brodt et al^157^ who demonstrated that multiple intravenous injections of liposomal **19** (2 µg/dose) administered either as a therapeutic or prophylactic/therapeutic treatment was equally effective in diminishing the number of hepatic metastases in H‐59 lung carcinoma‐bearing mice. On the contrary, liposomal **19** had no therapeutic activity when mice were inoculated with high tumor cell number indicating that immunotherapy with MDP analogs might only be effective when tumor burden is not too extensive. In another study, it was demonstrated that single intravenous injection of **19** (1 µg) incorporated into liposomes as well as free MDP (100 µg) significantly decreased the growth of hepatic metastases in M5076 reticulum cell sarcoma–bearing mice when used as prophylactic treatment, whereas the therapeutic treatment failed to inhibit the metastatic growth.^156^ The authors speculated that the lack of therapeutic activity was a result of defective Kupffer cell phagocytotic and/or migratory functions.

##### ImmTher

*N*‐acetylglucosaminyl‐*N*‐acetylmuramyl‐l‐Ala‐d‐isoGlu‐l‐Ala‐GDP also known as DTP‐GDP (**20**) is a lipophilic liposome‐encapsulated disaccharide tripeptide derivative of MDP (known under the brand name ImmTher) and a potent inducer of monocyte‐mediated cytotoxicity in vitro and in vivo.^159^, ^160^, ^161^ For example, in vitro study of Worth et al^159^ showed that **20** activated human monocytes to produce inflammatory cytokines (TNF‐α, IL‐1, IL‐6, IL‐8, IL‐12, macrophage chemotactic, and activating factor) and inhibit the growth of several human cell lines including Ewing's sarcoma (RD‐ES, SK‐ES‐1, and A4573‐EWS), osteosarcoma (SAOS‐2, MG‐63, and TE‐85), and melanoma (A375). The exact mechanism by which **20**‐stimulated monocytes affect tumor cells has not been fully elucidated but could, in addition to direct monocyte‐tumor cell contact, also include indirect activation of T cells and NK cells via an IL‐12–mediated mechanism.^159^

A phase I clinical trial was initiated to assess the toxicology and biological activity of ImmTher in patients with advanced colon carcinoma. ImmTher proved to be safe in humans up to a single dose of 1.2 mg/m^2^ and when given weekly for up to 6 months at doses 0.8 to 1 mg/m^2^. Toxic effects occurred at doses greater than 0.8 mg/m^2^ and included fever, chills, and hypotension. More importantly, ImmTher caused a regression of lung and liver metastases in three patients with metastatic colon carcinoma.^160^, ^161^ Due to the promising anticancer activity observed in preclinical and phase I clinical study ImmTher entered phase II clinical study to assess the 2‐year disease‐free survival of patients with high‐risk Ewing's sarcoma receiving vincristine, DOX, CTX, and dexrazoxane in the presence or absence of **20**.^162^ Since the lungs are common site of metastases in Ewing's sarcoma, the primary goal of **20** is to activate pulmonary macrophages to destroy residual tumor cells not eliminated by systemic chemotherapy (therapy with **20** is initiated after completion of primary therapy, such as surgery and radiotherapy). Nevertheless, **20** was designated as an orphan drug in the United States for indications including pulmonary and hepatic metastases in patients with colorectal adenocarcinoma, Ewing's sarcoma, and osteosarcoma.^163^

##### Dialkylmethyl β‐glycosides of MDP

Zemlyakov et al^87^ identified four lipophilic glycosides of MDP with symmetric secondary aliphatic alcohols as aglycones that demonstrated promising cytotoxic activities in vitro. In fact, the three most lipophilic compounds caused practically complete lysis of human erythroleukemia cells (K‐562) and evident cytotoxicity on blood mononuclear cells when used at their highest tested concentration (200 µg/mL). Furthermore, these three compounds were also strongly cytotoxic when tested on K‐562 and blood mononuclear cells at concentrations ranging from 2 to 20 µg/mL. It has been reported that the cytotoxic effect toward leukemia cells observed in the previous experiment resulted not only as a consequence of the direct cytotoxic activity of the tested compounds but also from their ability to activate NK cells in a blood mononuclear cell population. Moreover, the ability of compounds to stimulate the cytotoxic activity of NK cells correlates well with the increase in the number of aglycone carbon atoms. Thus, compound **21**, with the highest lipophilicity of the tested compounds, demonstrated the highest stimulation of NK cells.^87^

##### MDP‐C

Recently, MDP‐C (**22**) was identified as a new MDP analog that mediated its immunostimulating effect through macrophages and DCs.^90^ In a cell‐based assay, **22** stimulated the cytotoxic activity of murine macrophages against P388 leukemia cells resulting in an inhibitory rate of 71%. In comparison, romurtide (**11**) used as a positive control demonstrated a much lower inhibitory rate of 45%. Since compound **22** by itself was not cytotoxic to bone marrow–derived DCs, macrophages or P388 cells when tested under the same experimental conditions, it has been suggested that tumor growth inhibition was a result of macrophage activation by compound **22**.

#### 5.2.2 MDP conjugates

MDP is rapidly excreted from biological systems, resulting in a weaker stimulation of immune cells in vivo. As described earlier, its activity can be enhanced by introducing lipophilic moieties or by its incorporation into liposomes. Another mode of enhancing the biological activities includes the conjugation of MDP to macromolecules, such as IgG, IgM, BSA, fibronectin, cholesterol, and 10‐mer polyguanylic acid. As in the case of liposomes, biomolecules facilitate the transport of MDP and enable its phagocytosis by target cells, monocytes, and macrophages. Within these cells, molecules are released and then bind to NOD2 resulting in the activation of NOD2 signaling pathway. Alternatively, MDP has also been conjugated to small molecule drugs such as paclitaxel, batracylin, and acridine. In contrast to MDP‐biomolecule conjugates which mainly induce the antitumor activity of monocytes or macrophages, these conjugates induce anticancer activity either by direct cell killing or via the modulation of TME.

##### Conjugates with biomolecules

**Figure 6:**
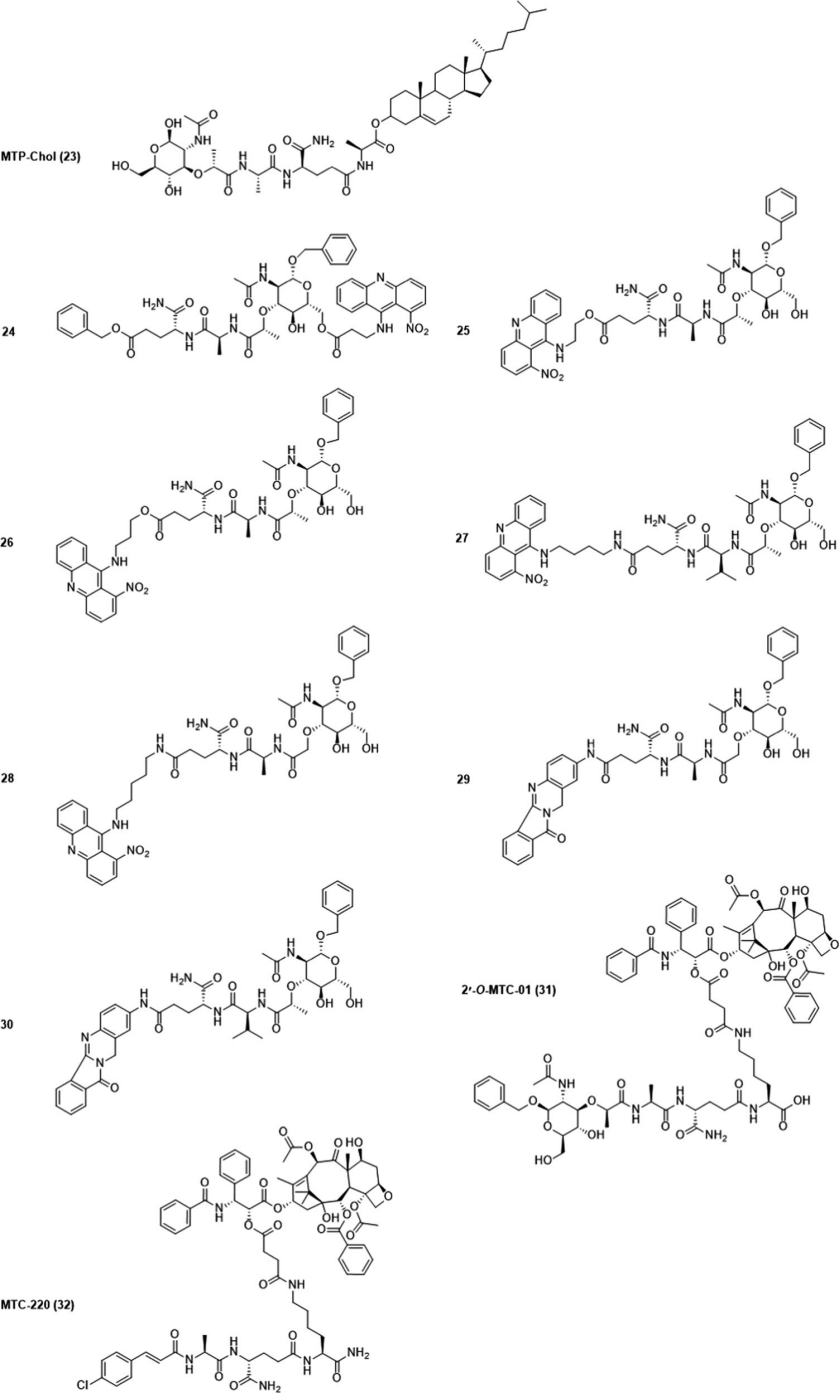
Muramyl dipeptide (MDP) conjugates with anticancer activity

The conjugation of MDP‐l‐Ala to cholesterol, an essential structural component of animal cell membranes, resulted in a new lipophilic MDP derivative MDP‐l‐Ala‐3‐*O*‐cholesterol (**23**; Figure 6) which showed promising macrophage‐mediated cytotoxicity in vitro when incorporated into liposomes. Philips et al^164^ demonstrated that **23** (1 µg/mL) incorporated into DSPC:PS (7:3 molar ratio) liposomes efficiently induced the cytotoxic activity of mouse peritoneal macrophages against P815 mastocytoma cells whereas treatment with free MDP at the concentration of 50 µg/mL had no effect. As opposed to the results obtained in mouse peritoneal macrophages, free MDP (10 µg/mL or greater) was capable of activating rat alveolar macrophages against B16‐BL6 melanoma cells, however, this activation was far more extensive when MDP or **23** were encapsulated into DSPC:PS liposomes. Comparison of relative activities of MDP, liposomal MDP and liposomal **23** in stimulating rat macrophage‐mediated cytotoxicity revealed that liposomal MDP and liposomal **23** were 880‐ and 7400‐fold more effective than free MDP, respectively.^164^ Results confirmed that encapsulation of MDP into liposomes indeed enhances macrophage‐mediated cytotoxicity when compared with that of free MDP. However, the retention of hydrosoluble MDP in liposomes is still poor resulting in loss of 90% of MDP.^164^ Conjugation to cholesterol improved the lipophilic character of **23** and facilitated its incorporation into phospholipid bilayers resulting in stable **23**‐containing liposomes with greatly improved macrophage‐activating properties as free or liposomal MDP. In addition to liposomal formulations, the effect of **23** incorporated into polymeric carrier systems such as nanocapsules was also studied, given the greater stability as well as improved characteristics following oral delivery.^165^ These studies showed that **23** incorporated into nanocapsules exhibited antimetastatic activity in a murine model of liver metastasis (histiocytosarcoma M5076 bearing mice), but only when administered as a prophylactic treatment.^165^, ^166^ Treatment of mice with nanocapsulated **23** given intravenously (5 µg/dose) or orally (50 µg/dose) twice a week beginning 2 days before M5076 tumor cell injection significantly decreased the number of metastases in the liver. Specifically, systemic and oral administrations resulted in 52% and 23% inhibition of metastasis in comparison to untreated controls, respectively. In contrast, the inhibition of metastasis was not observed in the absence of pretreatment.^165^ Results suggested that administration of **23** before tumor inoculation allows the circulating tumor cells to encounter activated macrophages. However, after the establishment of metastases in liver parenchyma, access by the activated liver macrophages is more restrained. In an attempt to enhance the antimetastatic activity of **23** in the liver, nanocapsulated **23** was tested in combination with nanocapsules containing indomethacin, a nonsteroidal anti‐inflammatory drug, based on the finding that liposomal indomethacin demonstrated antimetastatic activity in mice bearing 3LL Lewis lung carcinoma.^166^, ^167^ Two separate injections of nanoencapsulated **23** (5 µg/dose) and nanoencapsulated indomethacin (100 µg/dose) beginning 2 days before tumor cell injection resulted in enhanced antimetastatic activity which seemed to be additive.^166^ The exact mechanism of additive antimetastatic activity has yet to be elucidated but may, at least in the case of **23**, include activation of monocytes and macrophages. Attempts have also been made to improve the anticancer activity of MDP by binding it to various protein carriers, namely neoglycoproteins,^168^ antibodies,^169^ maleylated bovine serum albumin (MBSA),^170^ and gelatin,^171^ as well as to the nonproteinaceous carrier polyguanylic acid (MDP‐PolyG).^172^ Roche et al^168^ demonstrated that conjugation of MDP to neoglycoproteins enhanced the tumoricidal activity of macrophages, both in vitro and in vivo, thereby protecting the mice against metastatic growth. Moreover, they also found that MDP bound to IgM mAbs specific for L1210 leukemic cells (F_2_‐10‐23‐IgM) and for Lewis lung carcinoma 3LL cells (6B6‐IgM), activated thioglycolate‐elicited mouse peritoneal macrophages, in turn leading to a growth inhibitory effect in target cancer cells.^169^ Specifically, the coating of L1210 tumor cells with MDP‐F2‐10‐23‐IgM (10 µg/mL MDP bound to 200 µg/mL F2‐10‐23‐IgM) and incubated with macrophages resulted in 80% growth inhibition of L1210 tumor cells whereas comparable concentrations of free MDP resulted only in 5% to 10% tumor growth inhibition. Moreover, 3LL cells coated with MDP‐6B6‐IgM were even more efficient in activating macrophages. Only 5 µg/mL of MDP bound to 200 µg/mL of 6B6‐IgM resulted in 70% growth inhibition of 3LL cells.^169^ Results obtained in that study indicate the potential of mAbs to efficiently enhance the macrophage‐mediated cytotoxicity of MDP against cancer cells. Using this approach only macrophages inside or around the tumor would be activated leading to more specific antitumor activity and also less systemic activation of macrophages. Furthermore, Tabata et al^171^ studied four conjugates of MDP, namely MDP‐gelatin, MDP‐IgG, MDP‐fibronectin, and MDP‐BSA, in terms of their macrophage uptake as well as their ability to induce tumoricidal macrophages. To examine macrophage‐mediated cytotoxicity against Meth A fibrosarcoma (R1) cells, macrophages were isolated from the peritoneal cavity of mice who received one intraperitoneal injection of each MDP conjugate (containing 10 µg of MDP) or free MDP (10 µg). The order of the ability of conjugates to enhance macrophage activation was in good agreement with that for macrophage uptake and was highest in the case of MDP‐gelatin, followed by MDP‐IgG and MDP‐fibronectin, whereas MDP‐BSA, as well as free MDP, showed no activity.^171^ The highest efficacy, shown by the MDP‐gelatin conjugate, could be a consequence of the high specific affinity of gelatin for macrophages, whereas the absence of activity in the case of MDP‐BSA could be explained by the fact that it is difficult for BSA to be ingested by macrophages.^171^ Further experiments in mice bearing Meth A fibrosarcoma (R1) confirmed these findings. Administration of four intraperitoneal injections of MDP‐gelatin conjugate (containing 10 µg of MDP) into mice every third day after R1 tumor cell inoculation strongly suppressed the growth of R1 cells whereas free MDP (10 µg) showed no inhibitory activity under the same experimental conditions.^171^ More recently, Srividya et al^170^, ^172^ described scavenger receptor‐mediated endocytosis as a new option for delivery of MDP into macrophages and consequently treatment of cancer. MDP conjugated to MBSA or to polyguanylic acid (PolyG) was internalized by macrophages through scavenger receptor‐mediated endocytosis, which resulted in a 50‐ or 20‐fold higher cytotoxic activity against tumor cells, in comparison to that elicited by free MDP. It has been proposed that this type of MDP delivery activates tumoricidal activity of macrophages by triggering the secretion of cytokines (IL‐1, IL‐6, and TNF‐α) and other soluble mediators leading to final eradication of cancer cells.

##### Conjugates with small molecule drugs

Natural and synthetic acridines/acridones are known as potent cytotoxic agents but their clinical application is limited or has even been discontinued due to their severe side effects.^173^ Dzierzbicka et al^86^, ^174^ synthesized several series of conjugates of (nor)MDP with acridine and acridone derivatives to yield compounds with anticancer activity and improved pharmacological properties. Of all the synthesized compounds, the derivatives **24**, **25**, **26**, **27**, **28**, **29**, and **30** showed some promising results. Compound **24** stimulated the cytotoxic activity of NK cells derived from healthy and Ab melanoma–bearing animals, while compounds **25**, **26**, **27**, and **28** exhibited potent cytotoxic activity against several human cell lines.^86^, ^174^ Moreover, compounds **25**, **27**, and **28** were also active in vivo in the hollow fiber assay and compound **26** showed in vivo activity against UACC‐62 melanoma in mice.^86^, ^174^ Dzierbicka et al^175^ prepared a series of (nor)MDP analogues conjugated to a heterocyclic aryl amine batracylin at the carboxylic group of d‐isoGln at the C terminus of the peptide residue. Compounds **29** and **30** inhibited the proliferation in vitro of Ab melanoma cells, as well as demonstrating a prominent proapoptotic effect in WEHI 164 fibrosarcoma cells in the presence of immune cells.^176^ Furthermore, MDP analogs were also covalently linked to paclitaxel (PTX), one of the most widely used chemotherapeutic agent for treatment of various types of cancer. Several analogs (3′‐*N*‐MTC‐01, 2′‐*O*‐MTC‐01, and 7‐*O*‐MTC‐01) were prepared by coupling MDP analogs to 3′‐amino, 2′‐hydroxyl, or 7‐hydroxyl group of PTX. Among them, compound 2′‐*O*‐MTC‐01 (**31**) showed the most potent antitumor as well as immunostimulatory activity in vitro. Although compound **31** by itself demonstrated growth inhibitory effect against a panel of human cell lines with IC_50_ in the nM concentration range (1.3‐320 nM, 72 hours treatment), the tumor growth inhibition was not as efficient as that of PTX alone (0.3‐280 nM, 72 hours treatment). Further experiments in murine peritoneal macrophages demonstrated that compound **31** increased the expression and production of TNF‐α and IL‐12, particularly at a concentration of 5 µM or higher in a dose‐dependent manner. Interestingly, the ability of **31** to induce expression and production of TNF‐α and IL‐12 even surpassed that of PTX at a concentration of 5 µM.^177^ The ensuing in vivo experiments on mice bearing metastasis of LLC, however, showed that **31** was completely devoid of antimetastatic activity.^178^ To obtain analogs of MDP with antitumor and antimetastatic activities, compound **31** was further modified by replacing the muramic acid moiety by various aromatic groups, leading to the discovery of MTC‐220 (**32**).^178^ The results obtained by National Cancer Institute‐60 Human Tumor Cell Lines Screen showed that **32** inhibited growth of various tumor cell lines, with a mean GI_50_ (concentration of drug to cause 50% reduction in proliferation of cancer cells) of 22 nM. Its effectiveness was further confirmed in mouse xenograft models, where **32** effectively inhibited the growth of human breast (MDA‐MB‐231, MCF‐7) and lung cancers (H460, A549, and H1975). Moreover, **32** also exhibited antimetastatic activity in spontaneous metastasis model of LLC and highly invasive and metastatic 4T1 mammary carcinoma model. Specifically, multiple injections of **32** (10 mg/kg) administered daily for 15 days into mice with established LLC tumors resulted in statistically significant inhibition of tumor growth (33%) and lung metastasis (47%) in comparison to control group. Although similar tumor growth inhibition (26%) was observed when LLC bearing mice were treated with 6 mg/kg of PTX (equimolar dose of **32**), PTX had no effect on lung metastasis numbers in comparison to control group. The results obtained in a 4T1 mammary carcinoma model further confirmed the antitumor and antimetastatic activity of **32**. The significant tumor growth inhibition (approximately 30%) was observed when 4T1 bearing mice received multiple injections of **32** (5 mg/kg) or PTX (3 mg/kg) given daily for 28 days. As in the case of LLC, only **32** significantly reduced the number of lung metastases in a 4T1 mouse model of lung metastasis when compared with the control group. Detailed mechanistic studies suggested the connection between the antimetastatic activity of **32** and its ability to modulate inflammatory TME. It has been discovered that **32** suppressed the accumulation MDSCs in the spleen and bone marrow of 4T1 tumor‐bearing mice, while also repressing the expression of several metastasis‐promoting factors including TNF‐α, chemokine ligand 2 (CCL2), transforming growth factor (TGF) β, and matrix metalloproteinase (MMP) 9 in tumor tissue.^178^ These results are very encouraging since MDSCs play an important role in tumor progression and metastasis formation. Namely, MDSCs can increase production of MMP9 (involved in tumor angiogenesis promotion) or enhance tumor cell invasion and migration through the TGFβ pathway. The fact that **32** reduced the expression of TNF‐α is of particular interest since, in the majority of studies, MDP derivatives increased TNF‐α expression, in that way contributing to the anticancer effect. There are, however, reports of an ambiguous role of TNF‐α in cancer progression. Besides its anticancer activity, TNF‐α is also involved in the development of the tissue architecture necessary for tumor growth and metastatic dissemination, as well as in the induction of other cytokines, angiogenic factors, and MMPs, thus leading to the increased growth and survival of tumor cells.^179^ Although **32** exerts its antimetastatic activity through modulation of TME, there is still a lot unknown about the exact mechanism of this modulation. It has been speculated that **32** could also inhibit the TLR4 signaling pathway in cancer cells given the fact that the structure of **32** contains the PTX motif, which has been shown to bind to TLR4 receptors.

#### 5.2.3 Hydrophilic MDP derivatives

##### GMDP

**Figure 7:**
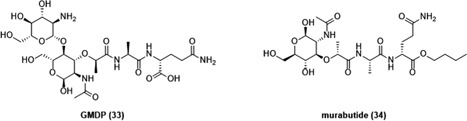
Hydrophilic muramyl dipeptide (MDP) derivatives with anticancer activity

GMDP (**33**; Figure 7) is a hydrophilic MDP derivative with an *N*‐acetylglucosamine residue attached to *N*‐acetylmuramic acid by a β(1,4)‐glycosidic bond. It is an effective immunomodulator, already marketed in Russia as Likopid, for combined treatment of various infectious diseases.^180^, ^181^ In addition to its immunomodulatory activities, **33** also mediates antitumor activity against several murine tumors including adenocarcinoma, LLC, melanoma, and sarcoma. The GMDP‐mediated growth inhibition of these tumors was, however, less than 60%.^182^ Furthermore, **33** also exhibits antimetastatic activity when given as prophylactic treatment. Experiments in LLC bearing mice showed that **33** reduced, 4.4‐ to 5.6‐fold, the number of metastases as well as their size (7‐10‐fold), as a result of GMDP‐mediated activation of NK cells.^183^ Since several studies revealed the ability of muramyl peptides to potentiate anticancer activity of therapeutic cytokines, the anticancer potential of **33** has been studied in combination with TNF‐α as well as other compounds includingTLR4 agonists (LPS, Lipid A analogs) and anticancer drugs. Shimizu et al studied the antitumor activity of **33** in a combination with low doses of LPS and its synthetic lipid A analogs A‐103 and 506, since these TLR4 agonists showed promising antitumor effect in several mouse tumor models. Two intravenous injections of A‐103 (50 µg) or **33** (10 µg) exhibited 43% or 52% inhibition of tumor growth rate, whereas a concomitant administration of A‐103 (100 µg) and **33** (10 µg) induced a significant 69% tumor growth inhibition when administered into Meth A fibrosarcoma bearing mice. In the same study it has also been shown that combinations of synthetic lipid A analogs (A‐103 and 506) (50 µg) or LPS (1‐10 µg) with **33** (10 µg) exhibited stronger inhibition of the tumor growth rate than when **33** was replaced by MDP (10 µg). Moreover, concomitant treatment with LPS and **33** resulted in three to four tumor‐free mice out of five.^184^ The exact mechanism underlying antitumor activity was not identified in this study, but could, at least in part, be attributed to macrophage activation and induction of TNF‐α secretion, as was observed in an experiment on mouse peritoneal exudate macrophages.^184^ Furthermore, **33** potentiated cytotoxic activity of TNF‐α against L929 murine fibrosarcoma cells. The synergistic effect was dose‐dependent and statistically significant when cells were treated for 24 hours with a combination of TNF‐α at a concentration range of 5 to 5000 U/mL and **33** at a concentration range of 0.014 to 140 µM. Moreover, treatment of L929 cells with a combination of GMDP (1.4 µM) and TNF‐α (10 U/mL) resulted in a greater number of apoptotic cells (43%) than that observed with TNF‐α alone (35%), indicating that **33** accelerated the TNF‐α–induced apoptosis.^185^ Furthermore, even better results in terms of cytotoxicity were observed when **33** was used in combination with TNF‐α and anticancer drugs such as actinomycin D (ActD) and CDDP in vitro.^185^, ^186^ For example, treatment of L929 cells with a TNF‐α in combination with ActD (4 µg/mL) resulted in 100% dead cells at 250 U/mL concentration, whereas in the presence of **33** (1.4 µM), a similar effect was observed even when TNF‐α and ActD were used at much lower 50 U/mL and 1 µg/L concentrations, respectively.^185^ In addition, **33** (1 µg/mL) also significantly potentiated the cytotoxic effect of TNF‐α (500 U/mL) and CDDP (3‐6 µM) against several other murine and human cell lines (L929, EAT, U‐93, and MCF‐7).^186^ Besides antitumor activity in vitro, **33** also exhibited antitumor activity in an in vivo setting. Specifically, it augmented the antitumor activity of TNF‐α and CDDP against Ehrlich ascites carcinoma and against melanoma B16 bearing mice.^187^ More importantly, treatment of mice with **33** at a dose as low as 0.05 µg/mouse decreased the toxicity of CDDP (40 µg/mouse)/TNF‐α (500 U/mouse) combination and normalized changes in hematological parameters (decreased lymphocytes, increased monocytes, and neutrophils) attributed to CDDP/TNF‐α treatment.^187^

Results obtained in these studies indicated that **33** synergizes with TNF‐α (and anticancer drugs) as well as augments anticancer effect of TNF‐α, one of the essential cytokines involved in regulation of cancer. Despite promising anticancer activity, the clinical application of TNF‐α is limited due to its high toxicity and deleterious side effects.^188^ Combination of **33** with TNF‐α and anticancer drugs reduced therapeutic doses of TNF‐α and anticancer drugs as well as augmented their therapeutic effect. This is particularly important since chemotherapeutics used in therapeutic doses, besides cancer cells, kill also normal rapid‐dividing cells such as the cells of bone marrow. The utilization of drugs characterized by synergistic effects, therefore, enables the reduction of therapeutically efficient doses, thereby decreasing the toxicity against normal cells.

##### Murabutide

Replacement of d‐isoGln with a d‐Gln‐n‐butyl‐ester residue in the peptide part of MDP afforded murabutide (**34**), another hydrophilic MDP derivative, that has similar adjuvant activity but lacks the pyrogenicity and toxicity of MDP.^189^, ^190^, ^191^, ^192^ Besides its adjuvant activity, **34** has also been shown to activate the immune system to fight cancer. Although in vast majority of studies MDP derivatives have been shown only to enhance anticancer activity of monocytes and macrophages, a study of Vidal et al^89^ showed that **34** triggered maturation and activation of monocyte‐derived immature DCs. **34**‐induced maturation of DCs was found to be beneficial since, in tumors, DCs are often found in an immature, immunosuppressive state and therefore unable to mount a proper immune response. Similarly, it has been proposed that the profile of cytokines secreted by **34**‐stimulated DCs including MIP‐1β, TNF‐α, IL‐10, and GM‐CSF, could be useful in mounting a strong immune response against tumors. In accordance with these results **34** at 10 µg/mL final concentration indeed significantly augmented the cytostatic activity of immature DCs against THP‐1 cancer cells.^89^ Moreover, treatment of DCs with **34** also resulted in an enhanced stimulatory capacity of DCs for both allogeneic and autologous T cells. These results revealed a great potential of **34** to be used as an adjuvant in DC‐based cancer vaccines.

Although **34** by itself has been shown to activate immune cells to fight cancer, an optimal activation was observed when **34** was used in combination with therapeutic cytokines IL‐2 and IFN‐α/β. In these studies, **34** synergized with IL‐2 or IFN‐α/β in vitro and in vivo as well as enhanced their biological activities. Specifically, in vitro experiments on human PBMCs revealed the synergistic activity of **34** and IL‐2 in inducing IL‐1β, IL‐12, IFN‐y, and CSFs. Moreover, in the same study it was demonstrated that the weak antitumor effect of IL‐2 in Meth A fibrosarcoma bearing mice was enhanced by concomitant administration of **34** (10 mg/kg, 5 or 3 times per week) and IL‐2 (5 × 10^6^ U/kg, 5 or 3 times per week) following 2 weeks of treatment. In fact, complete tumor regression was achieved in nearly 70% tumor‐bearing mice when they were treated with both compounds together.^193^ Importantly, concomitant treatment with both compounds was well‐tolerated since the net gain in body weight was not significantly different from that observed in the control group. The exact mechanism on how **34** potentiates antitumor activity of IL‐2 has not been elucidated in this study. On the basis of results of cytokine profile induced by a combination of **34** and IL‐2 in vitro, it was suggested that the antitumor effect may be due to induction of the Th1 cytokines IL‐12 and IFN‐y.^193^ In addition, stimulation of other effector mechanisms such as activation of NK cells killing cell activities could also be involved in potentiated antitumor effect. Similarly, synergistic antitumor activity was also observed when Meth A fibrosarcoma bearing mice were treated with **34** in combination with IFN‐α/β.^194^ Multiple injections of **34** (10 mg/kg) and IFN‐α/β (1.25 × 10^6^ U/kg), both given three times per week following 2 weeks of treatment into Meth A fibrosarcoma bearing mice resulted in almost 50% tumor‐free mice. In sharp contrast, treatment of tumor‐bearing mice with IFN‐α/β or **34** by themselves did not bring about a significant regression of tumor size.^194^ Collectively, the conducted studies show a limited efficacy and dose‐dependent toxicity of therapeutic cytokines in anticancer therapy. In our opinion, the utilization of safe immunomodulators such as compound **34**, capable of potentiating the anticancer activities of cytokines could, therefore, represent a significant advantage in the therapy of cancer. By employing this approach lower doses of therapeutic cytokines are needed for anticancer activity resulting in reduced toxicity, typically associated with high‐dose and long‐term administrations.

#### 5.2.4 Desmuramylpeptides

**Figure 8:**
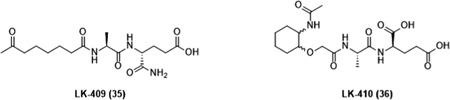
Desmuramylpeptides with antitumor and immunorestorative activity

The finding that the presence of the *N*‐acetylmuramyl moiety is not necessary for the immunomodulatory properties of MDP led to the design and synthesis of a new class of MDP derivatives, termed desmuramylpeptides.^195^, ^196^, ^197^, ^198^, ^199^, ^200^, ^201^, ^202^ Desmuramylpeptides are devoid of the *N*‐acetylmuramyl moiety and have therefore more lipophilic character than MDP. This class contains compounds that are able to enhance host defense against microbial infections as well as exhibit strong adjuvant activity and, even, remarkable antitumor potency.^202^ The latter was extensively studied using two nor‐MDP analogs, namely LK‐409 (**35**; Figure 8) and LK‐410 (**36**), in which the *N*‐acetylmuramyl moiety was replaced by the *N*‐(7‐oxooctanoyl) and *N*‐*trans*‐2‐((2′‐(acetylamino)cyclohexyl)oxy)acetyl groups, respectively.^203^, ^204^ It was demonstrated that treatment of SA‐1 fibrosarcoma bearing mice with multiple intraperitoneal injections of **35** (2.5 or 25 µg/dose) or **36** (25 µg/dose) administered 5 consecutive days after tumors achieved 35 mm^3^ in size resulted in a moderate but statistically significant inhibition of tumor growth measured as tumor growth delay (time required for the tumor to achieve a volume of 150 mm^3^). An antitumor effect was substantially augmented when either **35** or **36** were administered together with TNF‐α analog TNFNv3. The most significant tumor growth delay was seen when mice with established SA‐1 tumors received 2.5 µg of **36** (five injections in 5 consecutive days) and 5 × 10^5^ U of TNFNv3 (three injections given every second day), and was prolonged to 9.2 days when compared to untreated controls and 3.2 days when compared to TNFNv3 (5 × 10^5^ U/dose) treated mice. Remarkably, **36** also reduced the side effects of TNFNv3 in mice, resulting in lower body weight loss and better general conditions.^202^ Results indicate that desmuramylpeptides effectively potentiated the antitumor activity of TNF‐α analog, which is especially desirable since lower doses of compounds, in comparison to both compounds by themselves, are needed to achieve a similar antitumor effect. The exact mechanism on how these nor‐MDP analogs mediated antitumor activity has yet to be elucidated, but it most probably includes activation of macrophages. Moreover, **35** and **36** also demonstrated pronounced immunorestorative effects in vivo.^203^, ^204^ The effectiveness of **35** even surpassed that of romurtide (**11**) in restoring the activity of the immune response in tumor‐bearing mice and in immunocompromised animals evaluated in numerous in vitro and in vivo experiments.^204^ On the other hand, multiple injections of **36** at doses 10 and 100 mg/kg significantly increased the survival of mice suppressed by CTX and challenged with a suspension of *Candida albicans*.^203^ To evaluate the mechanism underlying its immunorestorative activity, **36** was further evaluated in several immunopharmacological models. It has been shown that **36** stimulated maturation of B cells as well as increasing the activity of B cells, T cells, and macrophages, but had no effect on cell counts.^203^ Since desmuramylpeptides have shown promising antitumor activity when used in combination with cytokines in addition to their ability to restore immune cell functions, in our opinion they could represent an excellent adjunct to current cancer therapy. Also, based on the observation that desmuramylpeptides can potentiate the antitumor activity of cytokines, it might be interesting to also test them in combination with other chemotherapeutics at low doses, potentially resulting in fewer side effects. Finally, desmuramylpeptides with immunorestorative activity could also restore the immune cell functions often impaired by chemotherapeutics, which is of particular importance, given the fact that the patients with the impaired immune system are more vulnerable to infection.

#### 5.2.5 SAR of MDP

**Figure 9:**
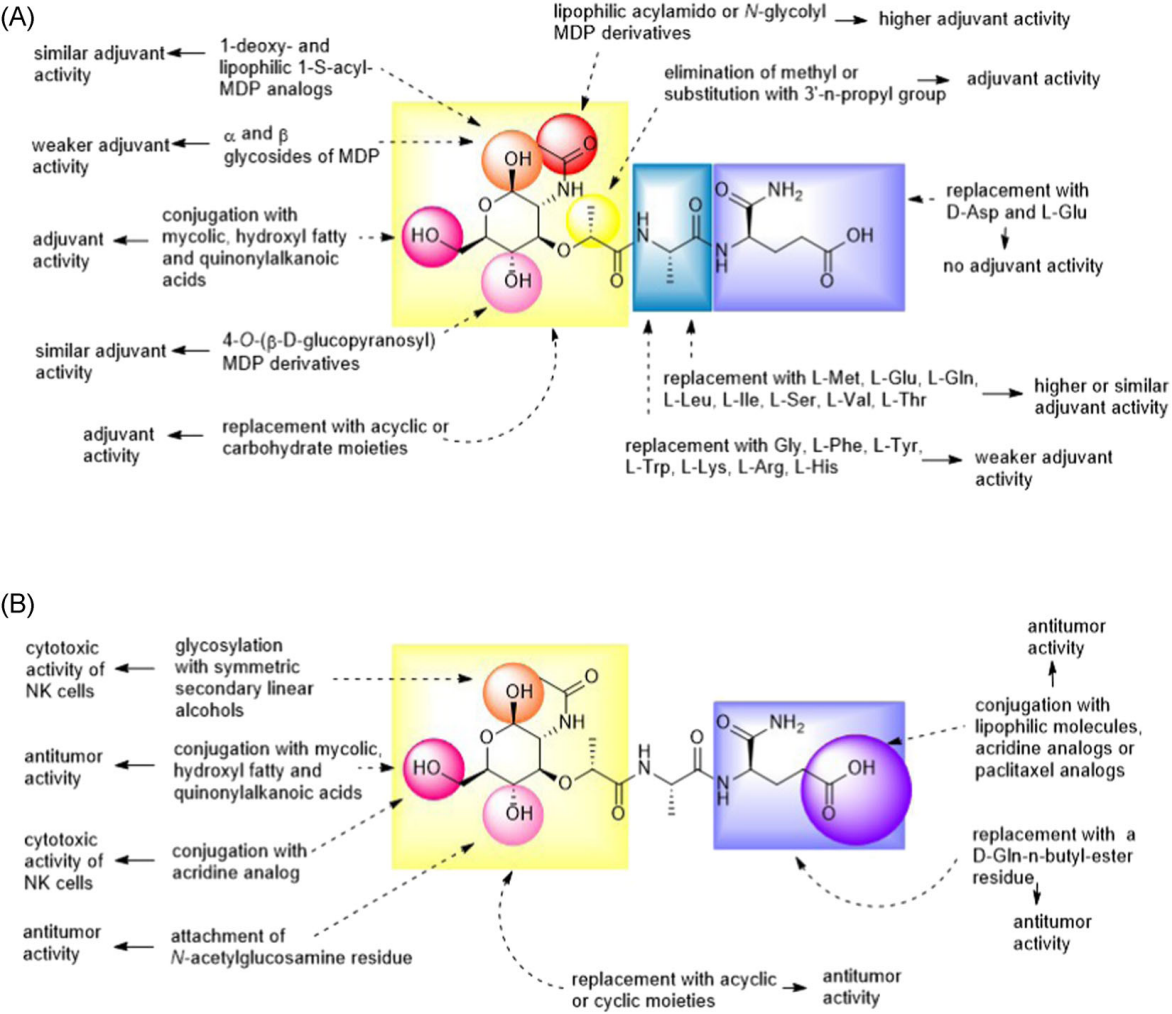
SAR of MDP. A, Modifications in MDP molecule in correlation with adjuvant activity and B, modifications in MDP molecule in correlation with antitumor activity. MDP, muramyl dipeptide; SAR, structure‐activity relationship [Color figure can be viewed at wileyonlinelibrary.com]

The structure of MDP in correlation with its adjuvant and antitumor activities has been widely studied. Investigations of their structure‐activity relationship (SAR) are summarized in Figure 9.

##### 5.2.5.1 Modifications of the peptide moiety

The l‐configuration of the first amino acid (l‐Ala) and the d‐configuration of the glutamic acid residue of the second amino acid (d‐isoGln) are essential for retaining or increasing the adjuvant activity of MDP.^205^ Substitution of l‐Ala with other amino acids generated MDP analogs with (i) similar or higher adjuvant activities (l‐methionine (l‐Met), l‐glutamic acid (l‐Glu), l‐glutamine (l‐Gln), l‐leucine (l‐Leu), l‐isoleucine (l‐Ile), l‐serine (l‐Ser), l‐valine (l‐Val), l‐threonine (l‐Thr)) or (ii) weaker (glycine (Gly), l‐phenylalanine (l‐Phe), l‐tyrosine (l‐Tyr), l‐tryptophan (l‐Trp), l‐lysine (l‐Lys), l‐arginine (l‐Arg), l‐histidine (l‐His)) than that of MDP.^206^, ^207^, ^208^ d‐isoGln (α‐amide) is an essential part of MDP, since its substitution with l‐Glu (γ‐amide) or d‐aspartic acid (d‐Asp) (side chain shortened by one methylene group) produces analogs devoid of adjuvant activity.^209^ In the context of the antitumor activity, conjugation of MDP at the C‐terminal end of the peptide part with lipophilic molecules such as stearic acid, dipalmitoyl phosphatidylethanolamine, GDP, cholesterol, *m*‐nitrocinnamic acid, acridine analogs, and paclitaxel analogs resulted in analogs with antitumor activity.

##### 5.2.5.2 Modifications of the carbohydrate moiety

Besides substitutions in the peptide portion, modifications can also be introduced into the carbohydrate moiety of MDP. The hydroxyl group at the C1 position can be eliminated, replaced by thiol, or substituted by α‐ or β‐glycosides without loss of adjuvant activity.^210^ 1‐deoxy and lipophilic 1‐*S*‐acyl analogs of MDP demonstrated strong adjuvant activity, closely similar to that of MDP,^211^, ^212^ while α‐ and β‐methyl glycosides showed weaker adjuvant activity.^213^ Moreover, dialkylmethyl β‐glycosides of MDP were found to stimulate cytotoxicity of NK cells.^87^ Furthermore, the acetamide group at the C2 position can be replaced by a hydroxyl, amino, methylamino, *N*‐methylacetamide or *N*‐glycolyl group.^210^, ^214^ Introduction of lipophilic acylamido or *N*‐glycolyl moieties into the structure of MDP led to analogs with enhanced adjuvant activity.^210^, ^214^ The chiral center of the lactic acid moiety at the C3 position appears to have a minimal effect on the biological activities of MDP. Elimination of the methyl group at the chiral center, or its substitution with 3′‐n‐propyl group, gives MDP analogs with adjuvant activity but lower toxicity than MDP.^210^ Substitution of the hydroxyl group at the C4 position is not that common, although some 4‐*O*‐(β‐d‐glucopyranosyl) derivatives of MDP demonstrated strong adjuvant activity, comparable to that of MDP.^215^, ^216^ Moreover, attachment of an *N*‐acetylglucosamine residue to the hydroxyl group at the C4 position gives **33**, a hydrophilic analog of MDP with antitumor activity. Substitution of the hydroxyl group at the C6 position by mycolic, hydroxyl fatty or quinonylalkanoic acids produced 6‐*O*‐acyl derivatives of MDP possessing significant adjuvant and antitumor activities.^142^, ^143^ Similarly, the 6‐*O*‐acyl derivative of MDP with acridine, compound **24**, stimulated the cytotoxic activity of NK cells derived from healthy and Ab melanoma–bearing animals.^86^ Moreover, some studies also showed that replacement of the *N*‐acetylmuramic acid in MDP with certain acyclic, cyclic, and carbohydrate moieties led to the retained immunomodulatory activity of MDP and, in some cases, even exhibited antitumor activities.^195^, ^196^, ^197^, ^198^, ^199^, ^200^, ^201^, ^202^

### 5.3 NOD1 and NOD2 antagonists

**Figure 10:**
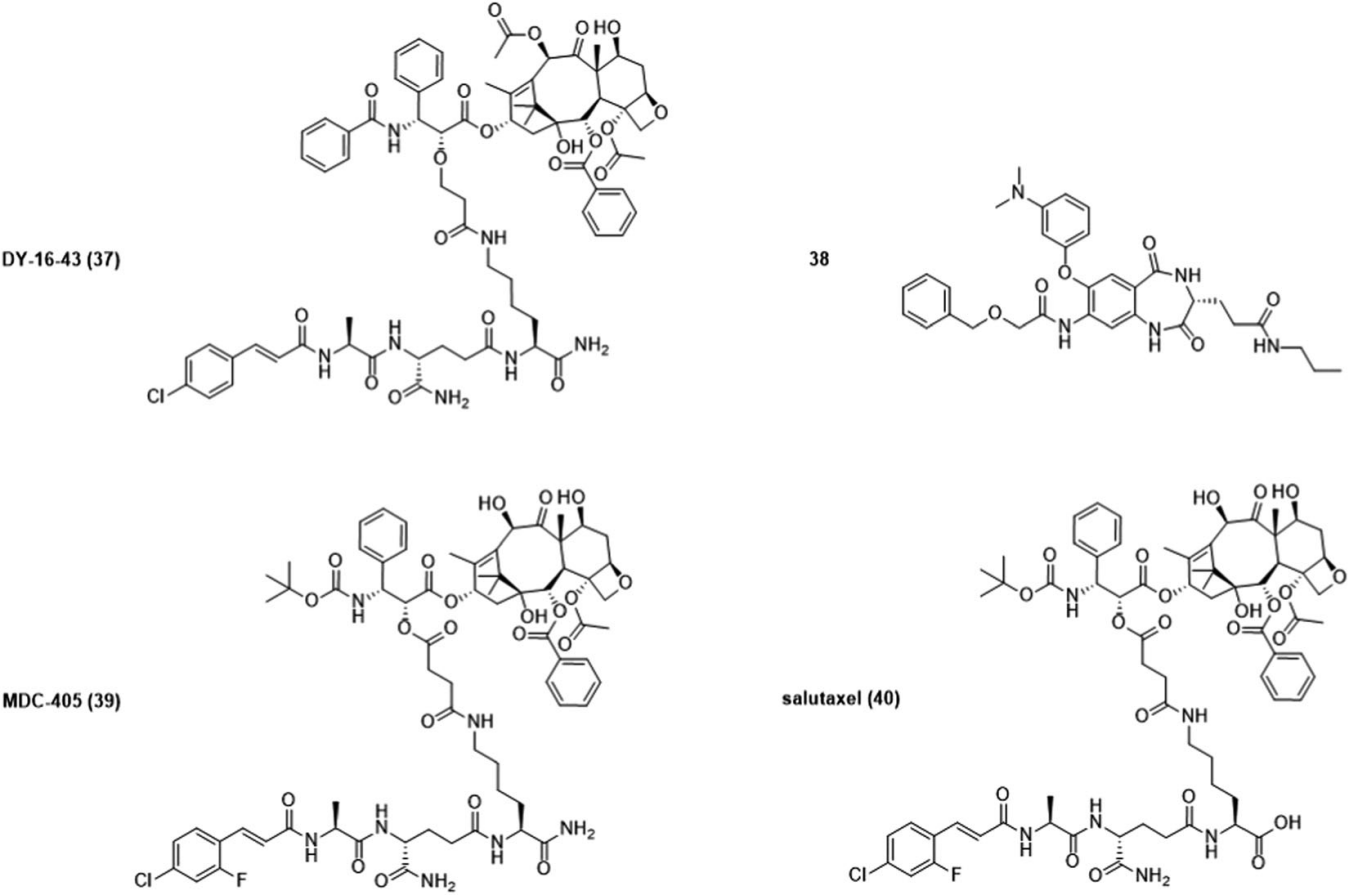
NOD1 and NOD2 antagonists with anticancer activity. NOD, nucleotide‐binding oligomerization domain

NOD antagonists have recently been recognized as potential anticancer agents with a unique mechanism of action. In sharp contrast to NOD agonists, which generally produce a pro‐inflammatory TME and enhance the ability of immune cells to fight cancer cells, it has been proposed that NOD antagonists mainly mediate their antitumor activity by preventing the formation of an inflammatory TME. Several compounds have so far been synthesized, among which DY‐16‐43 (**37**; Figure 10), **38**, MDC‐405 (**39**) and salutaxel (**40**) exhibit the most effective anticancer activity.

An NOD2 antagonist **37** (a noncleavable analog of **32**) markedly increased the therapeutic efficacy of PTX. It has been shown that concomitant treatment of LLC tumor‐bearing mice with multiple injections of PTX (12 mg/kg) and **37** (30 mg/kg) resulted, not only in the reduction of tumor weight but also in prevention of tumor metastasis. Surprisingly, treatment with **37** alone demonstrated no significant improvement in inhibition of tumor growth and prevention of metastasis. It was therefore proposed that, in mice, NOD2 was activated mainly by DAMPs generated as a result of the treatment with PTX and, in turn, resulted in TME remodeling, chemoresistance, and metastasis. Furthermore, it was suggested that **37** prevented the establishment of an inflammatory TME by blocking DAMPs and therefore sensitized the chemotherapeutic response of PTX.^217^ Recently, Wang et al^218^ identified a heterocyclic 1,4‐benzodiazepine‐2,5‐dione derivative **38** as a dual NOD1/NOD2 antagonist that, remarkably, enhanced the antitumor efficacy of PTX. Concomitant administration of multiple injections of **38** (20 mg/kg) and PTX (12 mg/kg) into mice beginning one day after LLC tumor cell inoculation resulted in a substantial tumor weight inhibition (67%) whereas treatment with PTX (34%) or **38** (10%) alone exerted no significant beneficial effect. Moreover, it has been shown that compound **38** could also decrease the mRNA levels of NOD1/2‐mediated IL‐6 and TNF‐α in human PBMC‐derived macrophages; further, it potently inhibited the NF‐κB and MAPK pathways. The exact mechanism underlying the observed antitumor activity of **38** has not so far been elucidated; however, it most probably involves the remodeling of TME as in the case of NOD2 antagonist **37**. Due to the promising results obtained by conjugates of PTX and MDP analogs, MDP analogs were further conjugated to docetaxel (DTX), another chemotherapeutic drug used in treating many cancers.^219^ Of all the synthesized DTX conjugates, compound **39** showed excellent results in preventing 4T1 breast tumor growth and metastasis in mice. Moreover, the beneficial effects of **39** have been associated with its ability to inhibit NOD1 signaling.^219^ Despite promising antitumor and antimetastatic activities in vivo, **39** has some unfavorable physicochemical characteristics such as low solubility in water. It has, therefore, been modified by replacing the amide in the C‐terminus with a carboxylic acid group, affording a new NOD1 antagonist **40**.^219^ In a preliminary screening, **40** inhibited growth of 14 human cancer cell lines of different origin with a mean GI_50_ of 16.3 nM after 72 hours treatment. Its effectiveness was further confirmed in mouse xenograft models, where **40** effectively inhibited the growth of human breast (MDA‐MB‐231), colon (HTC116), and lung (H1975, A549/T) cancers. Moreover, **40** also exhibited antimetastatic activity in a highly invasive and metastatic 4T1 mammary carcinoma model. Specifically, multiple injections of **40** (5, 10, or 20 mg/kg) given once per week for 15 days into mice with established LLC tumors demonstrated a substantial tumor growth inhibition in a dose‐dependent manner (12%, 37%, or 57%, respectively). Under the same experimental conditions, 5.7 mg/kg of DTX inhibited tumor growth only by 19%. Moreover, compound **40** also exhibited a significant antimetastatic effect at all doses used (5, 10, or 20 mg/kg) and was also significantly superior to DTX in the prevention of tumor metastasis when administered at doses of 10 or 20 mg/kg. In addition, compound **40** (10 mg/kg) was also tested in combination with DOX (4 mg/kg) and exhibited superior antitumor and antimetastatic effect in 4T1 carcinoma model in comparison to DTX (5.7 mg/kg)/DOX combination. Importantly, no significant body weight loss in mice was observed under the experimental conditions indicating that compounds were well tolerated at all tested concentrations. Mechanistically, **40** suppressed the accumulation of MDSC in the spleen of tumor‐bearing mice and decreased levels of several pro‐inflammatory molecules.^219^ As noted previously, MDSCs infiltrate the TME of developing tumors and promote their invasion and metastasis, most probably through the release of MMPs.^220^ Compound **40** was reported to significantly suppress MMP9 expression in the serum, spleen, and lungs of tumor‐bearing mice as well as to decrease the serum levels of tissue inhibitor of metalloproteinase (TIMP) 1 whose activity is also associated with the progression of several cancer types.^221^ Moreover, lung tissue derived from 4T1 tumor‐bearing mice treated with compound **40** demonstrated a significant decrease in the mRNA levels of prokineticin (PROK) 2, MMP8, S100 calcium‐binding protein A8 (S100A8) and S100A9, which might be also critically involved in metastasis formation.^219^ In addition, compound **40** also suppressed accumulation of neutrophils in the blood of 4T1 tumor‐bearing mice. This effect could be advantageous, since several studies have shown a correlation between elevated blood neutrophil count and poor clinical outcome in many cancers.^222^, ^223^ Moreover, it has been shown that dysfunctional neutrophils express reduced levels of NOD1 and that treatments that blocked the NOD1/NF‐κB pathway resulted in inhibition of neutrophil migration and their phagocytic killing capacity.^224^ Due to these findings, it has been proposed that compound **40** inhibits neutrophil recruitment by blocking the NOD1 pathway.

## 6 CONCLUSION

To date, a plethora of NOD ligands have been synthesized among which several compounds showed potent antitumor and even antimetastatic activity in numerous in vitro and in vivo studies (Supporting Information Table S1) as well as in clinical trials. Specifically, NOD2 agonist mifamurtide certainly represents the most important compound to have been granted with marketing authorization by the European Medicine Agency for treating osteosarcoma in combination with other chemotherapeutics, following complete surgical removal of a primary tumor. For a long time, only NOD agonists were considered as promising antitumor and antimetastatic compounds. Recently, a shift in this paradigm has occurred with the emerging knowledge that NOD antagonists could also facilitate the elimination of some cancers. Incidentally, the NOD1 antagonist salutaxel currently holds the status of an investigational new drug for cancer therapy. Furthermore, preclinical studies have also shown the great potential of NOD ligands for fighting cancer in a synergistic manner, in combination with chemotherapeutics, TLR ligands, cytokines, or when used as adjuvants in cancer vaccines.

To conclude, the innate immune receptors NOD1 and NOD2 constitute promising targets in cancer immunotherapy. It is, however, unlikely that NOD ligands alone could be sufficient for complete elimination of cancer, but their tumor‐suppressing capacities could be harnessed by introducing them as adjuncts to already existing cancer immunotherapies or to traditional cancer therapies such as chemotherapy and radiation.

## CONFLICTS OF INTEREST

The authors declare that there are no conflicts of interest.

## Supporting information

Supplementary information
