# Supplementary material for: Harnessing the untapped potential of nucleotide‐binding oligomerization domain ligands for cancer immunotherapy
Source: Med Res Rev. 2018 Dec 13;39(5):1447–84. doi: 10.1002/med.21557 (PMC6767550; doi:10.1002/med.21557)
Supplement: Supplementary file 1 — Supplementary information [file MED-39-1447-s001.docx]

**Table S1. Summarized anticancer activities of NOD ligands observed in *in vitro*/*in vivo* studies**

| **Compound** | **Activity** | **Description** | **Concentration/Dose/IC_50_/LC_50_** | ***in vivo*/*in vitro* model** | **Reference** |
| --- | --- | --- | --- | --- | --- |
| **NOD1 agonists** | | | | | |
| FK-156 (**3**) | Antitumor | Suppresses tumor growth in P388 tumor mouse model | 100 µg (i.t.) or 6 mg/kg (s.c.); 2 injections in 9 days after tumor cell inoculation | P388 lymphocytic leukemia bearing mice | ^105^ |
|  | Antitumor | Suppresses tumor growth in P388 tumor mouse model | 1-5 mg/kg (s.c.); 8 injections in 10 days after tumor cell inoculation | P388 lymphocytic leukemia bearing mice | ^105^ |
|  | Cytotoxic | Induces cytotoxic activity of resident peritoneal macrophages against P815 tumor cells | Minimum concentration of 10 µg/mL | P815 mouse mastocytoma cells | ^106^ |
| FK-565 (**4**) | Antitumor | Suppresses tumor growth in P388 tumor mouse model | 10-100 µg (i.t.) or 6 mg/kg (s.c.); 2 injections in 9 days after tumor cell inoculation | P388 lymphocytic leukemia bearing mice | ^105^ |
|  | Cytotoxic | Induces cytotoxic activity of resident peritoneal macrophages against P815 tumor cells | Minimum concentration of 10 µg/mL | P815 mouse mastocytoma cells | ^106^ |
|  | Antimetastatic | Administration in experimental lung metastasis model results in inhibition of lung metastasis formation | 1-10 mg/kg (s.c. or i.p.) administered prior to tumor cell inoculation | Experimental lung metastasis model (M109 lung carcinoma bearing mice) | ^106^ |
|  | Cytotoxic | Activates tumoricidal properties of mouse peritoneal macrophages against different tumor cells | 0.5-25 µg/mL (*in vitro* activation) | Syngeneic B16 mouse melanoma cells and xenogeneic tumor cells | ^107^ |
|  |  |  | 10 mg/kg (i.p.); multiple dose regimen (*in situ* activation) |  |  |
|  | Antimetastatic | Inhibits formation of lung metastasis in mouse | 1-10 mg/kg (s.c. or i.v.); 3 injections in 7 days after tumor cell inoculation | Mouse model of lung metastasis (B16 melanoma bearing mice) | ^107^ |
| FR-46758 (**5**) | Antitumor | Suppresses tumor growth in P388 tumor mouse model | 100 µg (i.t.) or 6 mg/kg (s.c.); 2 injections in 9 days after tumor cell inoculation | P388 lymphocytic leukemia bearing mice | ^105^ |
|  | Antitumor | Suppresses tumor growth in P388 tumor mouse model | 1-25 mg/kg (s.c.); 8 injections in 10 days after tumor cell inoculation | P388 lymphocytic leukemia bearing mice | ^105^ |
| FR-48217 (**6**) | Antitumor | Suppresses tumor growth in P388 tumor mouse model | 100 µg (i.t.) or 6 mg/kg (s.c.); 2 injections in 9 days after tumor cell inoculation | P388 lymphocytic leukemia bearing mice | ^105^ |
| FR-46091 (**7**) and FR-47920 (**8**) | Antitumor | Suppresses tumor growth in P388 tumor mouse model | 100 µg (i.t.); 2 injections in 9 days after tumor cell inoculation | P388 lymphocytic leukemia bearing mice | ^105^ |
| **NOD2 agonists** | | | | | |
| **Lipophilic MDP derivatives** | | | | | |
| Mifamurtide (MTP-PE (**10**) encapsulated into liposomes) | Cytotoxic | Activates monocytes of healthy donors to recognize and selectively kill tumor cells even when cocultured with normal cells | 0.25 μg (**10**) in liposomes (50 nmol) | Radiolabeled A375 human melanoma cells, HT-29 colon carcinoma cells, and Natusch human glioblastoma cells | ^114^ |
|  | Cytotoxic | Activates monocytes of healthy donors to become cytotoxic against A375 tumor cells | 0.5 μg (**10**) in liposomes (100 nmol) | Radiolabeled A375 human melanoma cells | ^117^ |
|  | Cytotoxic | Activates monocytes from renal cancer patients to become as cytotoxic as monocytes from healthy donors; For maximal cytotoxicity monocytes are activated with free **10** or mifamurtide in combination with IFN-γ (synergic effect) | 10 U/mL (IFN-γ) + 100 μg/mL (**10**) or 50 nM (mifamurtide) | Radiolabeled A375 human melanoma cells, SW626 human ovarian carcinoma cells and non-cultured fresh tumor cells | ^118,119^ |
|  | Cytotoxic | Activates monocytes from lung cancer patients to become as cytotoxic as monocytes from healthy donors | 500 nM | Radiolabeled A375 human melanoma cells | ^120^ |
|  | Cytotoxic | In a combination with IFN-γ synergistically activates tumoricidal properties in monocytes of healthy donors against A375 tumor cells | 1 or 10 U/mL (IFN-γ) + 50 nM (mifamurtide) | Radiolabeled A375 human melanoma cells | ^121^ |
|  | Antimetastatic | Administration as adjuvant treatment after surgical removal of primary tumor prolonged disease-free survival (DFS) and overall survival (OS) compared to placebo | 2mg/m^2^ (i.v.) twice weekly for 8 weeks | Dogs with spontaneous osteosarcoma | ^123^ |
|  | Antimetastatic | Adjuvant treatment after primary tumor resection improved OS compared with placebo when administered after, but not at the same time with CDDP | 2mg/m^2^ (i.v.) twice weekly for 8 weeks | Dogs with spontaneous osteosarcoma | ^122^ |
|  | Antimetastatic | Administration as adjuvant treatment after surgical removal of primary tumor prolonged DFS and OS compared with placebo when given concurrently with DOX/CTX | 2mg/m^2^ (i.v.) twice weekly for 8 weeks | Dogs with spontaneous splenic hemangiosarcoma | ^125^ |
|  | Antimetastatic | Administration as adjuvant treatment after excision of primary melanoma improved survival only in cases of low tumor burden | 10 μg twice weekly for 4 weeks | Mouse model of lymph node and lung metastasis (B16-BL6 melanoma-bearing mice) | ^126^ |
|  | Adjuvant | Induces antitumor immunity against SL2 lymphosarcoma cells when used as adjuvant in tumor vaccine | 20 µg in tumor vaccine; 2 (s.c.) injections in 10 and 20 days before tumor cell inoculation (prophylactic treatment) | SL2 lymphosarcoma bearing mice | ^132^ |
| Romurtide (**11**) | Antitumor | In combination with IFN-β significantly suppresses the growth of B16-F10 melanoma in mice | 100 ng-1 µg (**11**) + 10,000 U (IFN-β); 5 (i.d.) injections in 8-10 days after tumor cell inoculation | B16-F10 melanoma-bearing mice | ^89^ |
|  | Immunorestorative | Increases peripheral neutrophil, monocyte and platelet cell count in healthy monkeys | 1 mg (s.c.) administered for 10 consecutive days | Healthy cynomolgus monkeys (Macaca fascicularis) | ^137^ |
|  | Immunorestorative | Induces restoration of white blood cell count, mainly due to an increase in neutrophils | 100 µg (s.c) administered for 3 consecutive days | Mice with experimental leukopenia induced by CTX | ^138^ |
|  |  |  | 100 µg (s.c) administered for 8 consecutive days | Mice with experimental leukopenia induced by irradiation with X-rays |  |
|  | Antimetastatic | Inhibits lung metastasis of B16-BL6 melanoma in mice when used as prophylactic treatment | 100 µg (i.v., s.c., i.n.); 20 µg (s.c.); 1000 µg (o.) | Mouse model of lung metastasis (B16-BL6 melanoma-bearing mice) | ^142^ |
|  | Antimetastatic | Inhibits lung metastasis of colon 26-M3.1 carcinoma and liver metastasis of L5178Y-ML25 T lymphoma in mice when used as prophylactic treatment | 100 µg (s.c) | Mouse models of lung metastasis (26-M3.1 carcinoma-bearing mice) or liver metastasis (L5178Y-ML25 T lymphoma-bearing mice) | ^142^ |
|  | Antimetastatic | Inhibits lung metastasis of B16-BL6 melanoma when used as therapeutic treatment | 100 µg (s.c.); 5 injections after tumor cell inoculation | Mouse model of experimental and spontaneous lung metastasis (B16-BL6 melanoma-bearing mice) | ^142^ |
|  | Antitumor | Administration into mice results in tumoricidal activity of mouse peritoneal macrophages against B16-BL6 tumor cells as well as growth inhibitory effect by the sera towards B16-BL6 and L929 cells | 100 µg (s.c.) | Radiolabeled B16-BL6 mouse melanoma and L929 TNF-α-sensitive fibroblast cells | ^142^ |
|  | Antimetastatic | Administration reduces incidence of lung metastasis in hamsters with transplantable osteosarcoma | 50 μg (s.c.) administered every day for 3 weeks (**11** in solution) after tumor transplantation | Hamster’s osteosarcoma lung metastasis model | ^141^ |
|  |  |  | 20 μg (i.v.) administered twice a week for 3 weeks (**11** in liposomes) after tumor transplantation |  |  |
|  | Antimetastatic | Administration in hamsters after removal of primary tumor results in reduction of lung metastasis | 50 μg (s.c.) administered every day (**11** in solution) for 4 weeks after a hip joint amputation | Hamster’s osteosarcoma lung metastasis model | ^141^ |
|  |  |  | 20 μg (i.v.) administered twice a week (**11** in liposomes) for 4 weeks after a hip joint amputation |  |  |
| 6-*O*-mycoloyl-MDP (**12**), 6-*O*-nocardomycoloyl-MDP (**13**), 6-*O*-corynomycoloyl-MDP (**14**) and 6-*O*-mycoloyl-N-acetylmuramyl-l-Gly-d-isoGln (**15**) | Antitumor | Suppress tumor growth when administered into mice bearing fibrosarcoma | 100 µg (i.d.) administered in mixture with tumor cells | Meth A fibrosarcoma bearing mice | ^143,144^ |
| B30-MDP (**17**) | Adjuvant | Induces antitumor immunity against line 10 hepatoma or B-cell leukemia EN-L2C when used as adjuvant in tumor vaccine | 5-50 µg in tumor vaccine; 2 or 4 (i.d.) injections administered at 1 week intervals before tumor cell inoculation (prophylactic treatment) | Line 10 hepatoma or EN-L2C B-cell leukemia bearing strain-2 guinea-pigs | ^146,147^ |
|  | Adjuvant | Reduces metastases of L5178Y-ML25 mouse lymphoma when used as adjuvant in tumor vaccine | 100 µg in tumor vaccine; 1 injection administered before (prophylactic treatment) or after (therapeutic treatment) tumor cell inoculation | Murine liver and spleen metastasis model (L5178Y-ML25 lymphoma bearing mice) | ^148^ |
| Quinonyl-MDP-66 (**18**) | Antitumor | Suppresses tumor growth when administrated in mice bearing fibrosarcoma | 100 µg (i.d.) administered in mixture with tumor cells | Meth A fibrosarcoma bearing mice | ^149–151^ |
|  | Antitumor | Administration in strain-2 guinea pig tumor model results in suppression of line-10 hepatocarcinoma tumor growth | 0.1-0.4 mg (i.t.); 4 injections after tumor cell inoculation | Line-10 hepatocarcinoma bearing strain-2 guinea pigs | ^152^ |
|  | Immunorestorative | Administration into mice carrying 3LL tumors leads to restoration of depressed allogeneic cell-mediated cytotoxicity of spleen cells | 400 µg (i.p., i.v. or i.t.); 2 injections in 5 days after tumor cell inoculation | 3LL Lewis lung carcinoma bearing mice | ^153^ |
| MDP-GDP (**19**) encapsulated into liposomes | Cytotoxic | Induces alveolar macrophage cytotoxic activity against B16-BL6 melanoma cells when encapsulated into two different types of liposomes | 0.01-1 μg/mL (conventionally prepared liposomes; *in vitro* activation) | Radiolabeled B16-BL6 mouse melanoma cells | ^154^ |
|  |  |  | 0.009-18 μg/mL (freeze-dried liposomes; *in vitro* activation) |  |  |
|  |  |  | 10 μg/mouse (freeze-dried liposomes; *in situ* activation) |  |  |
|  | Antimetastatic | Reduces lung metastasis of B16-BL6 melanoma | 10 μg (i.v.) (freeze-dried liposomes;5 injections in 12 days after tumor cell inoculation) | Mouse model of lymph node and lung metastasis (B16-BL6 melanoma-bearing mice) | ^154^ |
|  | Antimetastatic | Exhibits activity against lung metastasis of B16 melanoma in mice when used as therapeutic treatment | 10 μg (i.v.); 5 injections in 13 days after tumor cell inoculation | Mouse model of lung metastasis (B16-BL6 melanoma bearing mice) | ^155^ |
|  | Cytotoxic | Activates tumoricidal activity of Kupffer cells against B16-F1 melanoma cells | 0.05-5 μg/mL (*in vitro* activation) | Radiolabeled murine B16-F1 melanoma cells | ^156^ |
|  |  |  | 0.1-1 μg/mouse (*in situ* activation) |  |  |
|  | Antimetastatic | Reduces liver metastasis of mice bearing B16-F1 melanoma when used as prophylactic or therapeutic treatment | 1 μg (i.v.); 1 injection before tumor cell inoculation (prophylactic treatment) | Mouse model of liver metastasis (B16-F1 melanoma-bearing mice) | ^156^ |
|  |  |  | 0.1-1 μg (i.v.); 4 injections in 14 days after tumor cell inoculation (therapeutic treatment) |  |  |
|  | Cytotoxic | Induces tumoricidal activity of mouse peritoneal macrophages and Kupffer cells against M5076 tumor cells | 1 μg/mL | Radiolabeled M5076 mouse histiocytic sarcoma cells | ^157^ |
|  | Antimetastatic | Reduces hepatic metastasis of mice bearing M5076 tumor cells when used as prophylactic treatment | 1 μg (i.v.); 1 injection | Mouse model of hepatic metastasis (M5076 histiocytic sarcoma ) | ^157^ |
|  | Antitumor | Activates tumoricidal activity of Kupffer cells against H-59 lung carcinoma | 2 μg/mouse (*in situ* activation) | Radiolabeled H-59 lung carcinoma cells | ^158^ |
|  | Antimetastatic | Reduces the number of hepatic micrometastases when used as prophylactic or therapeutic treatment | 2 μg (i.v.); 1 injection before and 4 injections after tumor cell injection (prophylactic/therapeutic treatment) | Mouse model of hepatic micrometastases (H-59 lung carcinoma bearing mice) | ^158^ |
|  |  |  | 2 μg (i.v.); 5 injections after tumor cell injection (therapeutic treatment) |  |  |
|  | Cytotoxic | Activates tumoricidal properties of murine Kupffer cells against B16-BL6 melanoma cells | 0.05-5 μg/mL (*in vitro* activation) | Radiolabeled B16-BL6 mouse melanoma cells | ^159^ |
|  |  |  | 0.1-1 μg/mouse (*in situ* activation) |  |  |
| ImmTher (**20**) | Cytostatic or cytotoxic | Stimulates cytostatic or cytotoxic activity of human monocytes against several tumor cells | n/a | Radiolabeled human Ewing's sarcoma (RD-ES, SK-ES-1, A4573-EWS), human osteosarcoma (SAOS-2, MG-63, TE-85) and human melanoma cells (A375) | ^160^ |
| **21** | Cytotoxic | Cytotoxicity toward K-562 tumor cells | 200 μg/mL | K-562 human erythroleukemia cells | ^88^ |
|  | Cytotoxic | Stimulates cytotoxic activity of NK cells towards K-562 tumor cells | 2-20 μg/mL | K-562 human erythroleukemia cells | ^88^ |
| MDP-C (**22**) | Cytotoxic | Activates mature murine macrophages to become cytotoxic against P388 tumor cells | 10 nM | P388 mouse leukemia cells | ^91^ |
| **MDP conjugates with biomolecules** | | | | | |
| MTP-chol (**23**) encapsulated into liposomes | Cytotoxic | Induces cytostatic activity of mouse peritoneal macrophages against P388 tumor cells | 1 μg/mL | Radiolabeled P815 mouse mastocytoma cells | ^165^ |
|  | Cytotoxic | Induces cytotoxic activity of rat alveolar macrophages against tumor cells | 0.0016-0.16 μg/mL (encapsulated in 8 nmol liposomes/culture), | Radiolabeled B16-BL6 mouse melanoma cells | ^165^ |
|  |  |  | 0.016-1.6 μg/mL (encapsulated in 80 nmol liposomes/culture) |  |  |
| MTP-chol (**23**) encapsulated into nanocapsules | Antimetastatic | Reduces the number of hepatic metastasis in mice bearing M5076 histiocytic sarcoma | 3-10 μg (i.v.) or 50-100 μg (o.); 1 dose administered before and 3 doses administered after tumor cell inoculation | Mouse model of liver metastasis (M5076 histiocytic sarcoma-bearing mice) | ^166^ |
|  | Antimetastatic | Reduces the number of hepatic metastasis in mice bearing M5076 tumor cells when used as prophylactic treatment | 5 μg (i.v.); 1 injection administered before (and 1 injection after) tumor cell inoculation | Mouse model of liver metastasis (M5076 histiocytic sarcoma-bearing mice) | ^167^ |
|  | Antimetastatic | In combination with nanocapsulated indomethacin reduces number of hepatic metastasis | 5 μg (**23**) + 100 μg (indomethacin); 1 injection before and 1 injection after tumor cell inoculation | Mouse model of liver metastasis (M5076 histiocytic sarcoma-bearing mice) | ^167^ |
| MDP-IgM (F_2_-10-23-IgM and 6B6-IgM) | Antitumor | MDP bound to IgM monoclonal antibodies specific for L1210 leukemic cells (F_2_-10-23-IgM) and for Lewis lung carcinoma 3LL cells (6B6-IgM), activates thioglycolate-elicited mouse peritoneal macrophages, which in turn led to a growth inhibitory effect in target cancer cells | 10 µg/mL MDP bound to 200 µg/mL F_2_-10-23-IgM (resulted in 80% growth inhibition) | L1210 mouse leukemia cells | ^170^ |
|  |  |  | 5 µg/mL MDP bound to 200 µg/mL 6B6-IgM (resulted in 70% growth inhibition) | 3LL mouse Lewis lung carcinoma cells |  |
| MDP-MBSA | Cytotoxic | Activates tumoricidal properties of J774A.1 macrophages against B16-F10 tumor cells and mouse peritoneal resident macrophages against L929 cells | 0.5-4 µg/mL (50 % lysis of the B16-F10 tumor cells) | Radiolabeled B16-F10 melanoma cells | ^171^ |
|  |  |  | 1-5 µg/mL (40 % inhibition of [3H]thymidine uptake by L929 cells) | Radiolabeled L929 fibroblast cells |  |
| MDP-gelatin, MDP-IgG, MDP-fibronectin and MDP-BSA | Cytotoxic | Activate mouse peritoneal macrophages to suppress the R1 tumor cell growth | 10 μg MDP in MDP-gelatin, MDP-IgG, MDP-fibronectin or MDP-BSA conjugate; 1 (i.p.) injection in mice | Mouse Meth A fibrosarcoma cells (R1) | ^172^ |
| MDP-gelatin | Antitumor | Strongly suppresses the growth of R1 tumor cells in mice | 10 μg MDP in MDP-gelatin conjugate; 4 (i.p.) injections in 7 days after tumor cell injection | Meth A fibrosarcoma (R1) bearing mice | ^172^ |
| MDP-PolyG | Cytotoxic | Activates tumoricidal properties of mouse peritoneal resident macrophages against L929 cells | 10 µg/mL (50% inhibition of [3H]thymidine uptake by L929 cells) | Radiolabeled L929 cells | ^173^ |
| **MDP conjugates with small molecule drugs** | | | | | |
| **24** | Cytotoxic | Induces cytotoxic activity of NK cells derived from healthy and Ab melanoma bearing hamsters against K562 cells | n/a | Radiolabeled K562 human erythroleukemic cell line | ^87^ |
| **25**, **26**, **27** and **28** | Cytotoxic | Exhibit cytotoxicity against several human cancer cell lines | MID log LC50 for compounds: -6.85 (**25**), -5.78 (**26**), -4.17 (**27**) and -5.82 (**28**) | Panel of human cancer cell lines | ^87,175^ |
| **25**, **27** and **28** | Antitumor | Exhibit antitumor activity against several human cancer cell lines cultivated in hollow fibers implanted in mice | n/a | Hollow fibers implanted in mice | ^87,175^ |
| **26** | Antitumor | Antitumor activity against UACC-62 melanoma in mice | 8.40 mg/kg (i.p) daily for 8 days | UACC-62 melanoma-bearing mice | ^175^ |
| **29** and **30** | Cytotoxic | Reduce proliferation of the tumor cell | 0.05 and 0.1 mg/mL | Ab melanoma cells | ^176^ |
|  | Cytotoxic | Induces apoptosis in WEHI 164 tumor cells | n/a | WEHI 164 mouse fibrosarcoma cells | ^177^ |
| 2′-*O*-MTC-01 (**31**) | Cytotoxic | Exhibits cytotoxicity against several human cancer cell lines | IC_50_ [nM] (72h):1.3 (KB), 3.0 (HeLa), 2.4 (BGC-823), 5.9 (A2780), 3.0 (MCF-7), 14.0 (PC3M ), 24.0 (KeTr3), 38 (HCT-8), 1.6 (A431), 170 (BEL-7402) | Human cancer cell lines: KB (head and neck cancer), HeLa (cervical cancer), BGC-823 (stomach cancer), A2780 (ovarian cancer), MCF-7 (breast cancer), PC3M (prostate cancer), KeTr3 (renal cancer), HCT-8 (colon cancer), BEL-7402 (hepatic cancer), and A431 (skin cancer) | ^178^ |
| MTC-220 (**32**) | Cytotoxic | Exhibits cytotoxicity against several human cancer cell lines | IC_50_ [nM] (72h): 31 (HCT-8), 0.0789 (Hela), 0.048 (A431), 0.146 (KB), 79 (A2780), 46 (KeTr3), 2.2 (PC3M), 4.67 (BGC-823) | Human cancer cell lines: HCT-8 (colon cancer), HeLa (cervical cancer), A431 (skin cancer), KB (head and neck cancer), A2780 (ovarian cancer), KeTr3 (renal cancer), PC3M (prostate cancer), and BGC-823 (stomach cancer) | ^179^ |
|  | Cytotoxic | Exhibits cytotoxicity against several human cancer cell lines | mean GI_50_ [nM]: approximately 22 nM | NCI60 human tumor cell lines screen | ^179^ |
|  | Cytotoxic | Inhibits the growth of several tumor types in mice | 10-20 mg/kg administered every day for 24 days (MDA-MB-231 xenograft model) | Mouse xenograft models using human breast (MDA-MB-231, MCF-7), ovarian (A2780, ES-2), and lung (H460, A549, H1975) tumor cell lines | ^179^ |
|  |  |  | 30 mg/kg administered every day for 12 days (MDA-MB-231, MCF7, A2780, ES-2, A549 and H1975 xenograft models); |  |  |
|  |  |  | 20 mg/kg administered every day for 24 days (H460 xenograft model) |  |  |
|  | Antitumor and antimetastatic | Inhibits tumor growth and decreases number of metastasis (nodule count) in lungs of mice | 10 mg/kg (i.p.) administered every day for 15 days | Mouse models of lung metastasis (LLC Lewis lung carcinoma bearing mice) | ^179^ |
|  |  |  | 2.5, 5, or 10 mg/kg (i.p.) administered every day for 28 days | Mouse models of lung metastasis (4T1 breast cancer bearing mice) |  |
| **Hydrophilic MDP derivatives** | | | | | |
| GMDP (**33**) | Antitumor | In combination with LPS or synthetic lipid A analogs (A-103 and 506) exhibits high tumor inhibition rate | 10 µg (**33**) + 100 µg (A-103) or 50 µg (506); 2 (i.v.) administrations in 9 days after tumor cell inoculation | Meth A fibrosarcoma bearing mice | ^185^ |
|  |  |  | 10 µg (**33**) + 1 or 10 µg (LPS); 1 administration on day 7 after tumor cell inoculation |  |  |
|  | Cytotoxic | Augments cytotoxic activity of TNF-α, ActD and combinations TNF-α/ActD or TNF-α/CDDP | 5–5,000 U/mL (TNF-α) + 0.014–140 µM (**33**) | L929 murine fibrosarcoma | ^186^ |
|  |  |  | 1 µg/mL (ActD) + 0.14–14µM (**33**) |  |  |
|  |  |  | 0,014-140 µM (**33**) + 0.25–50 U/mL (TNF-α) + 1 µg/mL (ActD) |  |  |
|  |  |  | 1.4µM (**33**) + 500 U/mL (TNF-α) + 1-90 µM (CDDP) |  |  |
|  | Cytotoxic | Augments cytotoxic activity of TNF-α/CDDP | 1.4µM (**33**) + 500 U/mL (TNF-α) + 1-10 µM (CDDP) | MCF7 human breast carcinoma | ^186^ |
|  | Cytotoxic | Augments cytotoxic activity of CDDP | 1.4µM (**33**) + 3-10 µM (CDDP) | U937 histiocytic lymphoma cells | ^186^ |
|  | Cytotoxic | Augments cytotoxic activity of TNF-α/CDDP | 1µg/mL (**33**) + 500 U/mL (TNF-α) + 3-6 µM (CDDP) | MCF-7, U-937, B16, and L-929 tumor cells, Ehrlich ascites carcinoma cells | ^187^ |
|  | Antitumor | Augments antitumor activity of TNF-α/CDDP in the mouse tumor models | 40 µg (CDDP) + 500 U (TNF-α) + 0.05 µg (**33**) administered (i.p.) weekly during 1 month period (optimal doses) | Ehrlich ascites carcinoma bearing mice | ^188^ |
|  | Antitumor | Augments antitumor activity of TNF-α/CDDP in the mouse tumor models | 40 µg (CDDP) + 500 U (TNF-α) + 0.05 µg (**33**); administered (s.c.) weekly during 3 months period (optimal doses) | B-16 melanoma bearing mice | ^188^ |
|  | Immunorestorative | Normalizes changes in hematological parameters (decreased lymphocytes, increased of monocytes and neutrophils) produced by the CDDP/TNF-α treatment | 0.05 µg (**33**) + 500 U (TNF-α) + 40 µg (CDDP) | F1[CBAxC57Bl/6j] mice | ^188^ |
| Murabutide (**34**) | Cytotoxic | Augments the ability of DCs to inhibit the growth of THP-1 cancer cells | 10 µg/mL | Radiolabeled THP-1 human monocytic leukemia cells | ^90^ |
|  | Antitumor | In combination with IL-2 exhibits synergistic antitumor activity in Meth-A sarcoma bearing mice | 10 mg/kg (**34**) + 5x10^6^ U/kg (IL-2); 5 or 3 (i.p.) injections weekly for 2 weeks | Meth-A fibrosarcoma bearing mice | ^194^ |
|  | Antitumor | In combination with IFN-α/β exhibits synergistic antitumor activity in Meth-A sarcoma bearing mice | 10 mg/kg (**34**) + 1.25x10^6^ U/kg (IFN-α/β); 3 (i.p.) injections weekly for 2 weeks | Meth-A fibrosarcoma bearing mice | ^195^ |
| **Desmuramylpeptides** | | | | | |
| LK-409 (**35**) | Antitumor | Demonstrated antitumor activity against SA-1 tumors in mice | 2.5-25 µg (i.p.); administered for 5 consecutive days in mice with established SA-1 tumors | SA-1 fibrosarcoma bearing mice | ^203^ |
|  | Antitumor | Augments antitumor effect of TNF-α analog TNFNv3 when administered into tumor bearing mice | 2.5-25 µg (**35**) administered (i.p.) for 5 consecutive days + 5x10^4^-5x10^5^ U(TNF-α) administered (peritumorally) three times every second day in mice with established SA-1 tumors | SA-1 fibrosarcoma bearing mice | ^203^ |
|  | Immunorestorative | Restores activity of immune response in tumor-bearing mice and immunocomprimised animals | n/a | Group of *in vitro* and *in vivo* tests | ^205^ |
| LK-410 (**36**) | Antitumor | Demonstrated antitumor activity against SA-1 tumors in mice | 25 µg (i.p.) administered for 5 consecutive days in mice with established SA-1 tumors | SA-1 fibrosarcoma bearing mice | ^203^ |
|  | Antitumor | Augments antitumor effect of TNF-α analog TNFNv3 when administered into tumor bearing mice | 2.5-25 µg (**36**) administered (i.p.) for 5 consecutive days + 5x10^4^-5x10^5^ U (TNF-α) administered (peritumorally) three times every second day in mice with established SA-1 tumors | SA-1 fibrosarcoma bearing mice | ^203^ |
|  | Immunorestorative | Protects mice against the immunosuppressive effect of CTX and increases the nonspecific resistance of mice against fungal infection | 10 and 100 mg/kg; 3 injections in 5 days administered in immunosuppressed mice infected with *Candida albicans* | Mice immunosuppressed with CTX and infected with *Candida albicans* | ^204^ |
| **NOD1 and NOD2 antagonists** | | | | | |
| DY-16-43 (**37**) | Antitumor and antimetastatic | In combination with PTX inhibits LLC tumor growth and reduces number of metastasis in lungs of mice | 30 mg/kg (**37**) administered every two days + 12 mg/kg (PTX) administered every 4 days; 4-week treatment after tumor cell injection | Mouse model of lung metastasis (LLC Lewis lung carcinoma-bearing mice) | ^218^ |
| **38** | Antitumor | In combination with PTX reduces LLC tumor size in mice | 20 mg/kg (**38**) administered daily + 12 mg/kg (PTX) administered every 4 day; 10-day treatment after tumor cell injection | LLC Lewis lung carcinoma-bearing mice | ^219^ |
| MDC-405 (**39**) | Antitumor and antimetastatic | Inhibits 4T1 tumor growth and decreases lung metastasis nodule counts in mice | n/a | Mouse model of lung metastasis (4T1 breast cancer-bearing mice) | ^220^ |
| Salutaxel (**40**) | Cytotoxic | Exhibits cytotoxicity against several human cancer cell lines | IC_50_ [nM] (72h): 50.00 (MDA-MB-231), 27.01 (MCF-7), 15.22 (MDA-MB-468), 2.27 (BT-474), 27.63 (A549), 10.12 (H460), 31.12 (H23), 1.27 (H522), 7.19 (OVCAR3), 10.11 (SF 295), 16.09 (COLO 205), 3.64 (KM-12), 12.87 (HT-29), 18.26 (MDA-MB-435) | Human cancer cell lines: MDA-MB-231 (breast cancer), MCF-7 (breast cancer), MDA-MB-468 (breast cancer), BT-474 (breast cancer), A549 (lung cancer), H460 (lung cancer), H23 (lung cancer), H522 (lung cancer), OVCAR3 (ovarian cancer), SF 295 (glioblastoma), COLO 205 (colon cancer), KM-12 (colon cancer), HT-29 (colon cancer), MDA-MB-435 (melanoma) | ^220^ |
|  | Antitumor | Inhibits the growth of several tumor types in mice | 5-10 mg/kg (i.v.) administered weekly for 3 weeks(MDA-MB-231 xenograft model) | Mouse xenograft models using human cancer cell lines: MDA-MB-231 (breast cancer), H1975 (lung cancer), HCT116 (colon cancer), and A549/T (lung cancer for paclitaxel resistance) xenograft mouse model | ^220^ |
|  |  |  | 3-9 mg/kg (i.v.) administered weekly for 3 weeks (H1975 xenograft model) |  |  |
|  |  |  | 5-20 mg/kg (i.v.) administered weekly for 3 weeks (HCT116 xenograft model) |  |  |
|  |  |  | 15 mg/kg (i.v.) administered weekly for 3 weeks (A549/T xenograft model) |  |  |
|  | Antitumor and antimetastatic | Inhibits tumor growth and lung metastasis formation in 4T1 tumor bearing mice | 10-20 mg/kg (i.v.) administered weekly for 4 weeks (antitumor), | Mouse model of lung metastasis (4T1 breast cancer-bearing mice) | ^220^ |
|  |  |  | 5-20 mg/kg (i.v.) administered weekly for 4 weeks (antimetastatic) |  |  |
|  | Antitumor and antimetastatic | In combination with DOX inhibits tumor growth and lung metastasis formation when administered into 4T1 tumor bearing mice | 10 mg/kg (**40**) + 4 mg/kg (DOX) (i.v.) administered weekly for 4 weeks | Mouse model of lung metastasis (4T1 breast cancer-bearing mice) | ^220^ |

ActD, actinomycin D; CDDP, cisplatin; CTX, cyclophosphamide; DC, dendritic cell; DFS, disease-free survival; DOX, doxorubicin; GDP, glycerol dipalmitate; GMDP, glucosaminyl muramyl dipeptide; IFN, interferon; i.d., intradermal administration; i.n., intranasal administration; i.p., intraperitoneal administration; i.v., intravenous administration; i.t., intratumoral administration; LPS, lipopolysaccharide; MBSA, maleylated BSA; MDP, muramyl dipeptide; MTP-chol, MDP-l-Ala-3-*O*-cholesterol; MTP-PE, muramyl tripepride phosphatidyl ethanolamine; n/a, not available; NK, natural killer; o., oral administration; OS, overall survival; PolyG, polyguanylic acid; PTX, pactitaxel; s.c., subcutaneous administration; TNF-α, tumor necrosis factor α
